# An updated checklist of the European Butterflies (Lepidoptera, Papilionoidea)

**DOI:** 10.3897/zookeys.811.28712

**Published:** 2018-12-31

**Authors:** Martin Wiemers, Emilio Balletto, Vlad Dincă, Zdenek Faltynek Fric, Gerardo Lamas, Vladimir Lukhtanov, Miguel L. Munguira, Chris A. M. van Swaay, Roger Vila, Albert Vliegenthart, Niklas Wahlberg, Rudi Verovnik

**Affiliations:** 1 UFZ – Helmholtz Centre for Environmental Research, Department of Community Ecology, Theodor-Lieser-Str. 4, 06120 Halle, Germany UFZ – Helmholtz Centre for Environmental Research Halle Germany; 2 Turin University, Department of Life Sciences and Systems Biology, via Accademia Albertina 13, I-10123 Torino, Italy Turin University Torino Italy; 3 Department of Ecology and Genetics, PO Box 3000, University of Oulu, 90014 Oulu, Finland University of Oulu Oulu Finland; 4 Biology Centre CAS, Branisovska 31, 370 05 Ceske Budejovice, Czech Republic Biology Centre CAS Ceske Budejovice Czech Republic; 5 Museo de Historia Natural, Universidad Nacional Mayor de San Marcos, Apartado 14-0434, Lima-14, Peru Universidad Nacional Mayor de San Marcos Lima Peru; 6 Department of Karyosystematics, Zoological Institute of Russian Academy of Sciences, Universitetskaya nab. 1, St. Petersburg 199034, Russia Zoological Institute of Russian Academy of Sciences St. Petersburg Russia; 7 Departamento de Biología, Universidad Autónoma de Madrid, c/ Darwin 2, 28049 Madrid, Spain Universidad Autónoma de Madrid Madrid Spain; 8 Dutch Butterfly Conservation, PO Box 506, 6700 AM Wageningen, The Netherlands Dutch Butterfly Conservation Wageningen Netherlands; 9 Institut de Biologia Evolutiva (CSIC-Universitat Pompeu Fabra), Passeig Marítim de la Barceloneta 37, 08003 Barcelona, Spain CSIC-Universitat Pompeu Fabra Barcelona Spain; 10 Lund University, Department of Biology, Sölvegatan 37, 223 62 Lund, Sweden Lund University Lund Sweden; 11 University of Ljubljana, Biotechnical Faculty, Department of Biology, Jamnikarjeva 111, 1000 Ljubljana, Slovenia University of Ljubljana Ljubljana Slovenia

**Keywords:** checklist, butterflies, Europe

## Abstract

This paper presents an updated checklist of the butterflies of Europe, together with their original name combinations, and their occurrence status in each European country. According to this checklist, 496 species of the superfamily Papilionoidea occur in Europe. Changes in comparison with the last version (2.6.2) of Fauna Europaea are discussed. Compared to that version, 16 species are new additions, either due to cryptic species most of which have been discovered by molecular methods (13 cases) or due to discoveries of Asian species on the eastern border of the European territory in the Ural mountains (three cases). On the other hand, nine species had to be removed from the list, because they either do not occur in Europe or lost their species status due to new evidence. In addition, three species names had to be changed and 30 species changed their combination due to new evidence on phylogenetic relationships. Furthermore, minor corrections were applied to some authors’ names and years of publication. Finally, the name *Polyommatusottomanus* Lefèbvre, 1831, which is threatened by its senior synonym *Lycaenalegeri* Freyer, 1830, is declared a *nomen protectum*, thereby conserving its name in the current combination *Lycaenaottomana*.

## Introduction

**Figure 1.**
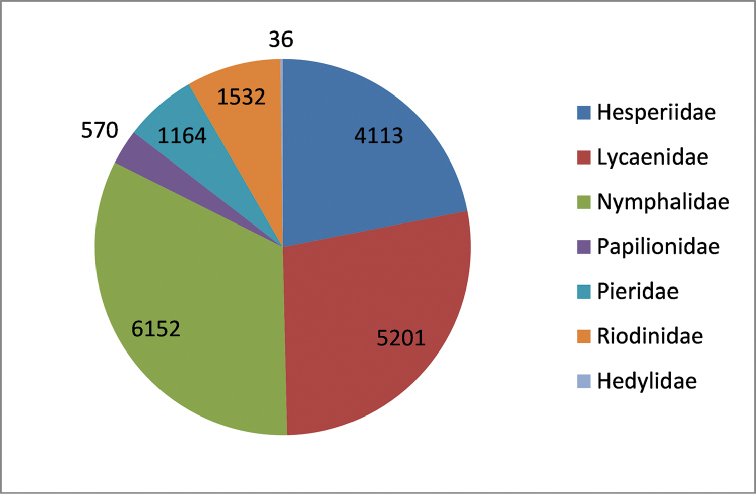
Global species richness of butterfly families.

Butterflies constitute one of the best-known groups of insects and have become important models to study speciation, community ecology, biogeography, climate change, and insect-plant interactions. With close to 19,000 described species [18,768 presumably valid species recorded by 2011; that figure is higher today, i.e., ca. 19,000 species], they represent about 12% of currently known species of Lepidoptera (Van Nieukerken et al. 2011). According to current molecular systematics (Mutanen et al. 2010; Heikkilä et al. 2012; Espeland et al. 2018), the single butterfly superfamily Papilionoidea comprises 7 families (Table 1, Fig. 1) and includes the Hesperiidae (skippers) and Hedylidae (moth butterflies). The skippers have previously been thought to represent the sister group to the butterflies and were often placed in a separate superfamily Hesperioidea, but the molecular results indicate that the family Papilionidae is the sister to the remaining butterflies, which also include the small Neotropical family Hedylidae with only 36 species. Apart from the latter family, all butterfly families are represented on all continents except Antarctica, although most species of Riodinidae are confined to the Neotropical Region. Butterfly diversity is particularly high in the tropics, especially the Neotropics, and only 496 species are found in Europe according to the present checklist.

**Table 1.** Family systematics of butterflies.

Superfamily Papilionoidea Latreille, [1802]	Genera*	Species*
Family Papilionidae Latreille, [1802]	32	570
Family Hedylidae Guenée, [1858]	1	36
Family Hesperiidae Latreille, 1809	570	4113
Family Pieridae Swainson, 1820	91	1164
Family Riodinidae Grote, 1895	146	1532
Family Lycaenidae [Leach], [1815]	416	5201
Family Nymphalidae Rafinesque, 1815	559	6152

* global number of genera and species according to van Nieukerken et al. (2011)

**Figure 2.**
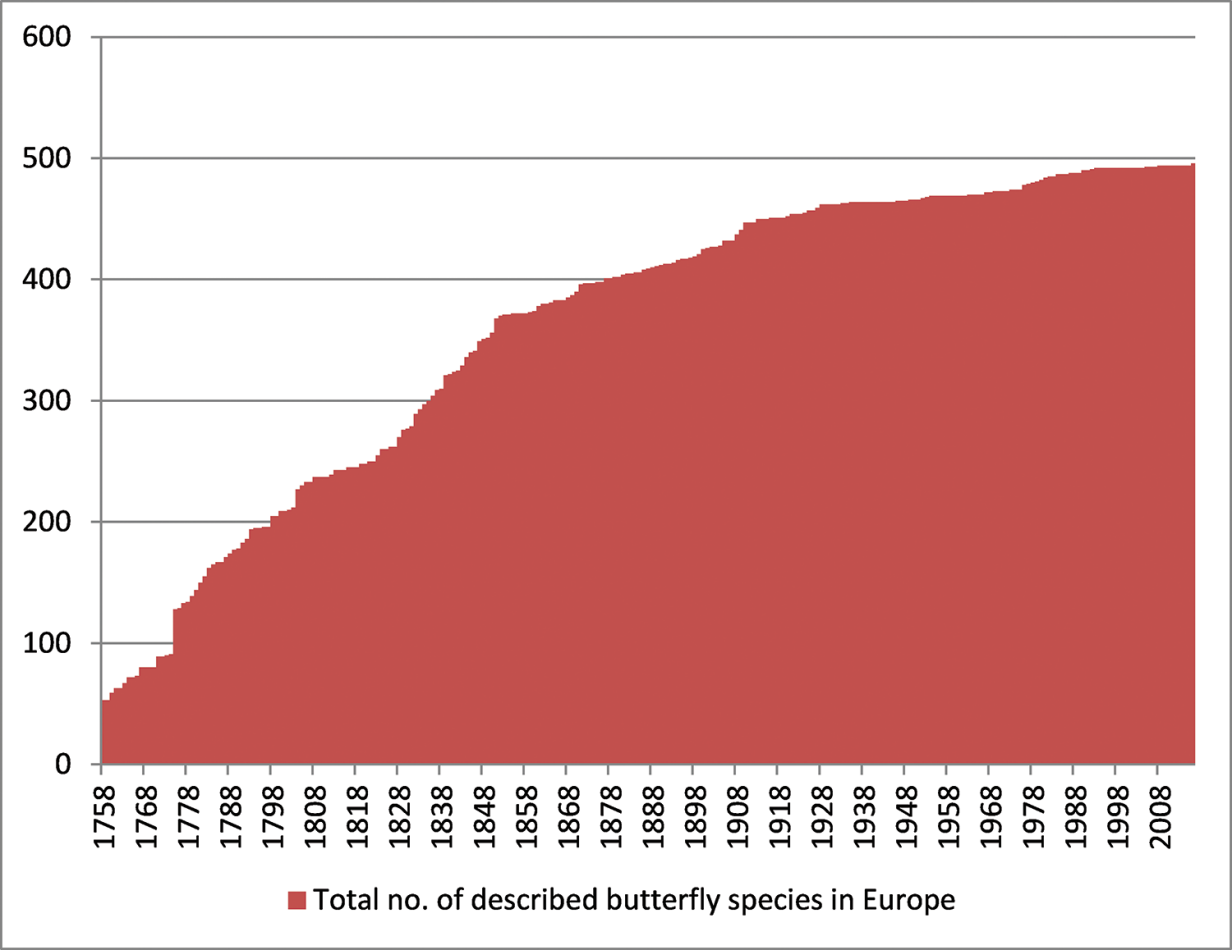
Cumulative number of described European butterfly species per year according to current taxonomy.

The taxonomy of butterflies started in 1758 with the Swedish naturalist Carl von Linné (Latinised to Carolus Linnaeus), who introduced binominal nomenclature and described the highest number of European butterfly species, all of them in a single genus *Papilio*. Seventy-one of them currently still hold the names given by Linné, albeit mostly in different genera. Other authors who described many new species during the 18^th^ century were the German entomologists Eugen Johann Christoph Esper and Jacob Hübner, the Danish entomologist Johann Christian Fabricius, as well as the Austrian lepidopterist Johann Ignaz Schiffermüller (the latter in an anonymous publication usually referred to as [Denis & Schiffermüller], but see Kudrna and Belicek (2005), Sattler and Tremewan (2009) and Kudrna (2015) for a controversial debate on this topic). By 1820, half of the European butterfly fauna had been validly described, and species were placed in a growing number of genera (starting with *Hesperia* Fabricius, 1793 as the second-named genus for the skippers). During the 19^th^ century, more than 60 European lepidopterists continued the inventory of Europe’s butterfly fauna, and the first overview of Palearctic butterflies (and other Lepidoptera) was published by Seitz (1907–1909). At that time, already 90% of Europe’s butterfly species had been described and the rate of newly discovered species slowed down (Fig. 2). Another milestone for butterfly research in Europe was the field guide of Higgins and Riley (1970), which included distribution maps of Western Palearctic butterflies, and led to a growing interest in butterflies across Europe. This field guide was also translated into other languages (e.g., German, French, and Spanish) and updated several times (most recently by Tolman and Lewington 2008). However, despite their somehow misleading titles, these guides excluded large parts of eastern Europe (i.e., Belarus, Ukraine, Moldova and most of Russia (apart from Kaliningrad enclave) and therefore all the species from the Ural mountains). The proliferation of butterfly field guides by various authors across Europe also led to an increasing confusion of butterfly nomenclature due to different taxonomic concepts. The first step to standardize European butterfly taxonomy and the precursor of our list was the book (and accompanying CD) by Karsholt and Razowski (1996). It constituted a country-level checklist of all European Lepidoptera, but excluding the Mid-Atlantic islands (i.e., Canary Islands, Madeira, and Azores) and contained 440 butterfly species. This book was also the basis for the list of Lepidoptera in the online database Fauna Europaea, a project under the auspices of the European Commission, which started in 2000 (De Jong et al. 2014) and aimed to provide checklists for all European animal species. This database, which went online on 16 December 2004, also included Cyprus and the Mid-Atlantic islands, which are hotspots of narrow endemics. At about the same time, the first distribution atlas of all European butterflies was published by Kudrna (2002), and finally a butterfly field guide appeared which covered most of the West Palearctic region including all of Europe (Tshikolovets 2011).

The last comprehensive update of the butterfly checklist in Fauna Europaea happened 7 years ago (Karsholt and Nieukerken 2011), and the checklist presented here was first developed as an update to the online database. Unfortunately, funding for Fauna Europaea was discontinued after the initial 4-years funding period and the outdated Fauna Europaea website was only saved due to the commitment of the Natural History Museum in Berlin that set up a new one. However, its functionality is still very limited and the update process severely hampered due to shortage of funding. For this reason, we decided to publish this updated distributional checklist in order to address the need of the lepidopterological community and the public at large. It intends to cover the significant progress in butterfly systematics and faunistics, which was brought about in particular by the advancement of molecular methods.

## Materials and methods

**Figure 3.**
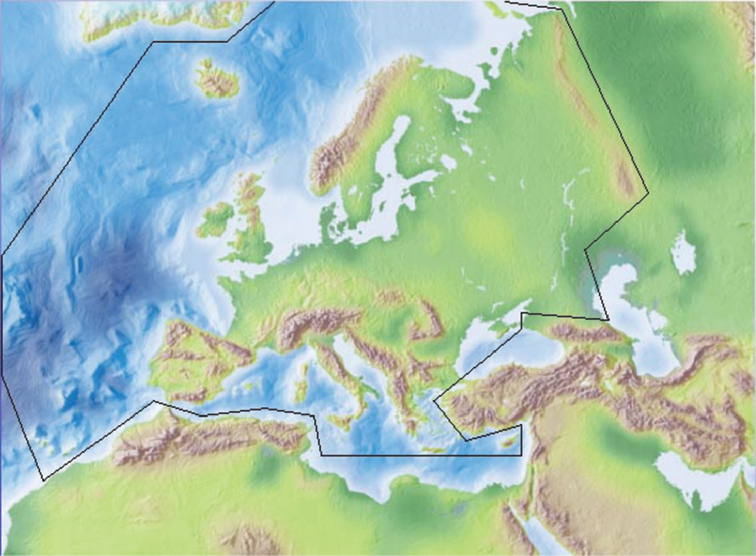
Boundaries of Europe according to Fauna Europaea (from de Jong et al. 2014).

This updated checklist is based on the last version of Fauna Europaea (2.6.2). This version is almost identical to the most recent Lepidoptera update in version 2.4 (online on 28 January 2011) but includes some emendations by the staff of the Fauna Europaea office in Berlin that had not been approved by the Lepidoptera group coordinators (Erik van Nieukerken and Ole Karsholt). The geographic area covered remains the same: It includes the European mainland to the eastern slopes of the Ural mountains, plus the Macaronesian islands (excluding the Cape Verde Islands) and Cyprus, with the Caucasus and western Kazakhstan excluded (Fig. 3). Included are the British Isles and all Mediterranean islands under European administration, as well as the Greek offshore islands along the Turkish coastline. Iceland has no native butterfly species. Distributional information is based on political units at country level as in Fauna Europaea, following the ISO-3166 code. However, with the exception of the Macaronesian Islands, the additional regional splits of several countries in Fauna Europaea (mainly for Russia and some island territories) were not adopted.

The following categories are used to explain the distribution:

A Absent (never recorded in the respective country or island group or only doubtful records)

P Present (native or well-established populations, including alien species such as the South African *Cacyreusmarshalli*)

P? Possibly present (recorded but continued presence doubtful; usually these are species with range limits near the border of the respective country)

M Regular migrant (species which has no permanent populations, e.g., because it cannot overwinter, but is observed almost every year; included are extinct species if they are still observed as regular migrants)

I Irregular vagrant (irregular vagrants or introductions which do not reproduce or only irregularly, including temporary or recently established populations)

Ex Regionally extinct (native species which have become extinct, even though vagrants might be seen occasionally)

It should be noted that the “Extinct” category is used in a rather strict sense, in line with the IUCN Guidelines which demand that exhaustive surveys have been undertaken to prove that ‘there is no reasonable doubt that the last individual has died’. In some cases, this has led to species being recorded as “Present”, even though they are most probably extinct, e.g., *Coliasmyrmidone* in Austria (no proof for more than 25 years; H. Höttinger, pers. comm.). In addition, some of the national Red List Assessments are already outdated, even though attempts have been made to update those. An example for an update is the status of the Madeiran endemic *Pieriswollastoni*, whose last reliable record is from 1986. It was classified as “Critically Endangered (Possibly Extinct)” in its last Red List assessment (Van Swaay et al. 2010), but is now classified as “Extinct”, because extensive surveys in recent years have failed to prove its continued presence. This is the only European butterfly species which is known to have become globally extinct in historical times.

According to the concept of Fauna Europaea, changes were only carried out if supported by newly published research. This restriction helps to stabilize nomenclature, but can also lead to inconsistent results, e.g., due to the retention of some weakly differentiated taxa, whose species status is questionable, but for which no new published evidence is available. Potential examples in our list are *Lysandracaelestissima* (Verity, 1921), *Polyommatusnephohiptamenos* (Brown & Coutsis, 1978), *Hipparchianeapolitana* (Stauder, 1921), *Hipparchiasbordonii* Kudrna, 1984, *Satyrusvirbius* Herrich-Schäffer, 1844, and *Pierisbalcana* Lorković, 1969.

The main criterion whether to include or exclude a species taxon based on new (and possibly contradictory) publications was evidence for species status from at least two character sets, e.g., mitochondrial as well as nuclear DNA, or differences in morphology and karyology.

Nomenclatural changes are annotated with reference to the sources and strictly follow the last (fourth) edition of the International Code of Zoological Nomenclature (ICZN 1999). This includes the controversial article 34.2, which mandates that »the ending of a Latin or Latinised adjectival or participial species-group name must agree in gender with the generic name with which it is at any time combined«. Due to its linguistic complexity, this rule has led to many wrong or ambiguous decisions and causes additional instability of nomenclature each time a species name is transferred to another genus. Therefore a majority of lepidopterists, including the group editors of Fauna Europaea, have decided to ignore this rule and use the original spelling instead (de Jong et al. 2014). Difficulties with the gender agreement rule in Lepidoptera are as old as binominal nomenclature, because there is not even an agreement about the gender of the genus *Papilio*. Therefore Carl von Linné used nouns as species names and avoided the use of adjectives (Welter-Schultes 2013). However, for easy reference to Fauna Europaea and other databases, we also list the original ending and compiled a comprehensive list of original combinations, using various sources such as the LepIndex (Beccaloni et al. 2003), PESI (2018), FUNET (Savela 2018) and Tshikolovets (2011). In case of doubts or discrepancies, the original publications were checked as well.

In a few cases, necessary changes due to new nomenclatural findings have not been carried out yet, because they would result in the replacement of a well-established name by an (almost) unknown synonym. Such cases should be referred to the International Commission on Zoological Nomenclature for ruling, and changes implemented only after a decision has been made by the Commission. One such case is the well-established name *Parnassiusphoebus*, which has turned out to represent another Asian *Parnassius* species which is currently known as *Parnassiusariadne* (Lederer, 1853) (see Hanus and Thèye 2010) and would thus need to be replaced. After the first attempt to preserve this name (Balletto and Bonelli 2014) failed (ICZN 2017), a second proposal has recently been submitted to the Commission (Lukhtanov et al. in press). According to article 82.1 of the code, prevailing usage has to be maintained until the case has been decided by the Commission.

An exceptional case which would cause a large number of changes in the names of Lepidoptera are many of the names published by [Denis & Schiffermüller] (1775) which are lacking a sufficient description, but have already been used for a very long time. In accordance with the opinion of the Fauna Europaea editorial team, we have not replaced these names. The effect on butterfly taxonomy would be rather marginal, however, because only one butterfly species would have to change its name (*Nymphalisvaualbum* to *Nymphalisl-album* (Esper, 1781)) and five others only their authorship, see Kudrna and Belicek (2005). We are looking forward to a decision of the ICZN to solve this matter (see Kudrna 2015).

Another case concerns the genus name *Muschampia* Tutt, 1906 (type species: *Papilioproto* Ochsenheimer, 1808; currently known as *Muschampiaproto* (Ochsenheimer, 1808)), which appears to be a subjective synonym of the genus name *Sloperia* Tutt, 1906 (type species: *Hesperiapoggei* Lederer, 1858; currently known as *Muschampiapoggei* (Lederer, 1858)). Both genus names were published in the same paper and Hemming (1967) was the first to note that *Sloperia* should have precedence over *Muschampia*, because Warren (1926) as the first reviser chose *Sloperia*. However, the name *Muschampia* has remained in prevailing use during the last 90 years and, in addition, there is evidence from molecular data (Wiemers et al. unpublished) that the current classification of the species presently placed in the genera *Carcharodus* and *Muschampia* needs to be substantially revised. However, molecular data are still missing for most of the (mainly Asian) species currently placed in *Muschampia*, and therefore we suggest to postpone a rearrangement until better data become available.

Finally, one of us (GL) discovered that *Polyommatusottomanus* Lefèbvre was published in 1831 (and not in 1830) and therefore has to be regarded as a subjective junior synonym of *Lycaenalegeri* Freyer, 1830. This would mean that the well-established name of the species currently known as *Lycaenaottomana* (Lefèbvre, [1831]) would need to be changed to a name which has not been used for this species during the past century. However, according to article 23.9.1 of the Code, the prevailing usage must be maintained when the senior synonym (i.e., *legeri* Freyer) has not been used as a valid name after 1899 (article 23.9.1.1), and the junior synonym has been used, as its presumed valid name, in at least 25 works, published by at least ten authors during the last 50 years and encompassing a span of not less than ten years (article 23.9.1.2). In our opinion, the condition of article 23.9.1.1 applies in this case, and evidence that the conditions of article 23.9.1.2 are met, are given in Appendix 1 herein. Therefore, we regard the name *Lycaenalegeri* Freyer as invalid and qualified as a *nomen oblitum* and declare the name *Lycaenaottomana* Lefèbvre as valid and qualified as a *nomen protectum*, which has precedence over the former as long as both names are thought to represent subjective synonyms.

## Results and discussion

**Figure 4.**
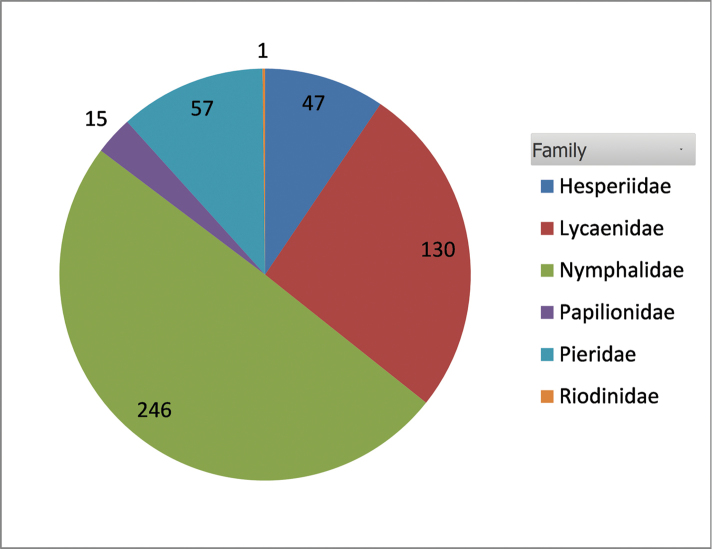
Species richness of butterfly families in Europe.

The updated species list of European butterflies includes 496 species, which belong to 110 genera in 21 subfamilies and six families (Tables 2 and 4; Fig. 4). A list of main authors with some additional data is given in Table 5. An electronic version of the checklist that includes a country-based distributional checklist is found in Suppl. material 1, Suppl. material 2.

**Table 2.** Updated checklist of the butterflies of Europe.

Taxon	Original combination	Notes
** Papilionidae **		
** Papilioninae **		
*Iphiclidespodalirius* (Linnaeus, 1758)	* Papilio podalirius *	
*Iphiclidesfeisthamelii* (Duponchel, 1832)	* Papilio feisthamelii *	1
*Papilioalexanor* Esper, 1800	* Papilio alexanor *	
*Papiliomachaon* Linnaeus, 1758	* Papilio machaon *	
*Papiliohospiton* Gené, 1839	* Papilio hospiton *	2
** Parnassiinae **		
*Parnassiusmnemosyne* (Linnaeus, 1758)	* Papilio mnemosyne *	
*Parnassiusphoebus* (Fabricius, 1793)	* Papilio phoebus *	
*Parnassiusapollo* (Linnaeus, 1758)	* Papilio apollo *	
*Archonapollinus* (Herbst, 1798)	* Papilio apollinus *	
*Zerynthiacerisy* (Godart, [1824])	* Thais cerisy *	
*Zerynthiacretica* (Rebel, 1904)	* Thais cerisyi cretica *	
*Zerynthiacaucasica* (Lederer, 1864)	* Thais cerisyi caucasica *	
*Zerynthiarumina* (Linnaeus, 1758)	* Papilio rumina *	
*Zerynthiapolyxena* ([Denis & Schiffermüller], 1775)	* Papilio polyxena *	
*Zerynthiacassandra* (Geyer, [1828])	* Papilio cassandra *	3
** Hesperiidae **		
** Heteropterinae **		
*Heteropterusmorpheus* (Pallas, 1771)	* Papilio morpheus *	
*Carterocephalussilvicola* (Meigen, 1829)	* Hesperia silvicola *	
*Carterocephaluspalaemon* (Pallas, 1771)	* Papilio palaemon *	
** Hesperiinae **		
*Pelopidasthrax* (Hübner, [1821])	* Gegenes thrax *	
*Borboborbonica* (Boisduval, 1833)	* Hesperia borbonica *	
*Gegenespumilio* (Hoffmansegg, 1804)	* Papilio pumilio *	
*Gegenesnostrodamus* (Fabricius, 1793)	* Hesperia nostrodamus *	
*Ochlodessylvanus* (Esper, 1777)	* Papilio sylvanus *	
*Hesperiacomma* (Linnaeus, 1758)	* Papilio comma *	
*Thymelicuschristi* Rebel, 1894	* Thymelicus christi *	
*Thymelicusacteon* (Rottemburg, 1775)	* Papilio acteon *	
*Thymelicushyrax* (Lederer, 1861)	* Hesperia hyrax *	
*Thymelicussylvestris* (Poda, 1761)	* Papilio sylvestris *	
*Thymelicuslineola* (Ochsenheimer, 1808)	* Papilio lineola *	
** Pyrginae **		
*Spialiaphlomidis* (Herrich-Schäffer, 1845)	* Hesperia phlomidis *	
*Spialiasertorius* (Hoffmansegg, 1804)	* Hesperia sertorius *	
*Spialiatherapne* (Rambur, 1832)	* Hesperia therapne *	
*Spialiarosae* Hernández-Roldán, Dapporto, Dincă, Vicente & Vila, 2016	* Spialia rosae *	4
*Spialiaorbifer* (Hübner, [1823])	* Papilio orbifer *	
*Carcharodustripolinus* (Verity, 1925)	* Erynnis alceae tripolina *	5
*Carcharodusalceae* (Esper, 1780)	* Papilio alceae *	
*Muschampiacribrellum* (Eversmann, 1841)	* Hesperia cribrellum *	
*Muschampiatessellum* (Hübner, [1803])	* Papilio tessellum *	
*Muschampiaproto* (Ochsenheimer, 1808)	* Papilio proto *	
*Carcharoduslavatherae* (Esper, 1783)	* Papilio lavatherae *	
*Carcharodusorientalis* Reverdin, 1913	* Carcharodus orientalis *	
*Carcharodusfloccifera* (Zeller, 1847)	* Hesperia floccifera *	
*Carcharodusstauderi* Reverdin, 1913	* Carcharodus stauderi *	
*Carcharodusbaeticus* (Rambur, 1839)	* Spilothyrus baeticus *	
*Erynnistages* (Linnaeus, 1758)	* Papilio tages *	
*Erynnismarloyi* (Boisduval, 1834)	* Thanaos marloyi *	
*Pyrgusmalvoides* (Elwes & Edwards, 1897)	* Hesperia malvoides *	
*Pyrgusmalvae* (Linnaeus, 1758)	* Papilio malvae *	
*Pyrguscarthami* (Hübner, [1813])	* Papilio carthami *	
*Pyrgussidae* (Esper, 1784)	* Papilio sidae *	
*Pyrguscentaureae* (Rambur, 1839)	* Hesperia centaureae *	
*Pyrguscacaliae* (Rambur, 1839)	* Hesperia cacaliae *	
*Pyrgusandromedae* (Wallengren, 1853)	* Syrichtus andromedae *	
*Pyrgusserratulae* (Rambur, 1839)	* Hesperia serratulae *	
*Pyrgusarmoricanus* (Oberthür, 1910)	* Syrichthus armoricanus *	
*Pyrgusalveus* (Hübner, [1803])	* Papilio alveus *	
*Pyrguswarrenensis* (Verity, 1928)	* Hesperia warrenensis *	
*Pyrgusfoulquieri* (Oberthür, 1910)	* Syrichthus alveus foulquieri *	6
*Pyrgusonopordi* (Rambur, 1839)	* Hesperia onopordi *	
*Pyrguscarlinae* (Rambur, 1839)	* Hesperia carlinae *	
*Pyrguscirsii* (Rambur, 1839)	* Hesperia cirsii *	
*Pyrguscinarae* (Rambur, 1839)	* Hesperia cinarae *	
** Pieridae **		
** Dismorphiinae **		
*Leptideaduponcheli* (Staudinger, 1871)	* Leucophasia duponcheli *	
*Leptideamorsei* (Fenton, 1882)	* Leptosia morsei *	
*Leptideajuvernica* Williams, 1946	* Leptidea sinapis juvernica *	7
*Leptideasinapis* (Linnaeus, 1758)	* Papilio sinapis *	
*Leptideareali* Reissinger, 1990	* Leptidea sinapis reali *	
** Coliadinae **		
*Gonepteryxrhamni* (Linnaeus, 1758)	* Papilio rhamni *	
*Gonepteryxcleobule* (Hübner, [1831])	* Anteos cleobule *	8
*Gonepteryxcleopatra* (Linnaeus, 1767)	* Papilio cleopatra *	
*Gonepteryxmaderensis* C. Felder, 1862	* Gonopteryx cleopatra maderensis *	
*Gonepteryxfarinosa* (Zeller, 1847)	* Rhodocera farinosa *	
*Catopsiliaflorella* (Fabricius, 1775)	* Papilio florella *	
*Coliashyale* (Linnaeus, 1758)	* Papilio hyale *	
*Coliasalfacariensis* Ribbe, 1905	* Colias hyale alfacariensis *	
*Coliasphicomone* (Esper, [1780])	* Papilio phicomone *	
*Coliasaurorina* Herrich-Schäffer, 1850	* Colias aurorina *	
*Coliaschrysotheme* (Esper, [1781])	* Papilio chrysotheme *	
*Coliaserate* (Esper, [1805])	* Papilio erate *	
*Coliascrocea* (Geoffroy, 1785)	* Papilio croceus *	5, 9
*Coliasmyrmidone* (Esper, [1781])	* Papilio myrmidone *	
*Coliascaucasica* Staudinger, 1871	* Colias myrmidone caucasica *	
*Coliaspalaeno* (Linnaeus, [1760])	* Papilio palaeno *	10
*Coliastyche* (Böber, 1812)	* Papilio tyche *	
*Coliashecla* Lefèbvre, 1836	* Colias hecla *	
** Pierinae **		
*Colotisevagore* (Klug, 1829)	* Pontia evagore *	
*Aporiacrataegi* (Linnaeus, 1758)	* Papilio crataegi *	
*Pontiachloridice* (Hübner, [1813])	* Papilio chloridice *	
*Pontiacallidice* (Hübner, [1800])	* Papilio callidice *	
*Pontiaedusa* (Fabricius, 1777)	* Papilio edusa *	
*Pontiadaplidice* (Linnaeus, 1758)	* Papilio daplidice *	
*Pieriskrueperi* Staudinger, 1860	* Pieris krueperi *	
*Pierisbrassicae* (Linnaeus, 1758)	* Papilio brassicae *	
*Pieriswollastoni* (Butler, 1886)	* Ganoris wollastoni *	
*Pierischeiranthi* (Hübner, [1808])	* Papilio cheiranthi *	
*Pierisrapae* (Linnaeus, 1758)	* Papilio rapae *	
*Pierismannii* (Mayer, 1851)	* Pontia mannii *	
*Pierisergane* (Geyer, [1828])	* Papilio ergane *	
*Pierisbryoniae* (Hübner, [1806])	* Papilio bryoniae *	
*Pierisnapi* (Linnaeus, 1758)	* Papilio napi *	
*Pierisbalcana* Lorković, [1969]	* Pieris balcana *	11
*Euchloetagis* (Hübner, [1804])	* Papilio tagis *	
*Euchloeeversi* Stamm, 1963	* Euchloe belemia eversi *	
*Euchloegrancanariensis* Acosta, 2008	* Euchloe belemia grancanariensis *	
*Euchloehesperidum* Rothschild, 1913	* Euchloe belemia hesperidum *	
*Euchloebelemia* (Esper, 1800)	* Papilio belemia *	
*Euchloeinsularis* (Staudinger, 1861)	* Anthocharis tagis insularis *	
*Euchloecrameri* Butler, 1869	* Euchloe crameri *	
*Euchloesimplonia* (Freyer, 1829)	* Pontia simplonia *	
*Euchloeausonia* (Hübner, [1804])	* Papilio ausonia *	
*Euchloecharlonia* (Donzel, 1842)	* Anthocharis charlonia *	
*Euchloepenia* (Freyer, 1851)	* Pontia penia *	
*Euchloebazae* Fabiano, 1993	* Euchloe charlonia bazae *	
*Zegrispyrothoe* (Eversmann, 1832)	* Pontia pyrothoe *	
*Zegriseupheme* (Esper, [1804])	* Papilio eupheme *	
*Anthochariseuphenoides* Staudinger, 1869	* Anthocharis euphenoides *	
*Anthochariscardamines* (Linnaeus, 1758)	* Papilio cardamines *	
*Anthocharisgruneri* Herrich-Schäffer, 1851	* Anthocharis gruneri *	
*Anthocharisdamone* Boisduval, 1836	* Anthocharis damone *	
** Riodinidae **		
** Nemeobiinae **		
*Hamearislucina* (Linnaeus, 1758)	* Papilio lucina *	
** Lycaenidae **		
** Lycaeninae **		
*Lycaenadimorpha* (Staudinger, 1881)	* Polyommatus dimorphus *	5, 12
*Lycaenahelle* ([Denis & Schiffermüller], 1775)	* Papilio helle *	
*Lycaenaalciphron* (Rottemburg, 1775)	* Papilio alciphron *	
*Lycaenathetis* Klug, 1834	* Lycaena thetis *	
*Lycaenathersamon* (Esper, 1784)	* Papilio thersamon *	
*Lycaenadispar* ([Haworth], 1802)	* Papilio dispar *	
*Lycaenahippothoe* (Linnaeus, [1760])	* Papilio hippothoe *	10
*Lycaenacandens* (Herrich-Schäffer, 1844)	* Polyommatus candens *	
*Lycaenaottomana* (Lefèbvre, [1831])	* Polyommatus ottomanus *	5, 13
*Lycaenableusei* (Oberthür, 1884)	Polyommatus xanthe f. bleusei	
*Lycaenaphlaeas* (Linnaeus, [1760])	* Papilio phlaeas *	10
*Lycaenavirgaureae* (Linnaeus, 1758)	* Papilio virgaureae *	
*Lycaenatityrus* (Poda, 1761)	* Papilio tityrus *	
**Aphnaeinae**		
*Cigaritisacamas* (Klug, 1834)	* Lycaena acamas *	14
** Theclinae **		
*Theclabetulae* (Linnaeus, 1758)	* Papilio betulae *	
*Favoniusquercus* (Linnaeus, 1758)	* Papilio quercus *	
*Laeosopisroboris* (Esper, [1793])	* Papilio roboris *	15
*Tomaresballus* (Fabricius, 1787)	* Papilio ballus *	
*Tomaresnogelii* (Herrich-Schäffer, 1851)	* Thecla nogelii *	
*Tomarescallimachus* (Eversmann, 1848)	* Lycaena callimachus *	
*Callophrysavis* Chapman, 1909	* Callophrys avis *	
*Callophryssuaveola* (Staudinger, 1881)	* Thecla suaveola *	
*Callophrysrubi* (Linnaeus, 1758)	* Papilio rubi *	
*Callophryschalybeitincta* Sovinsky, 1905	* Callophrys rubi chalybeitincta *	
*Neolycaenarhymnus* (Eversmann, 1832)	* Lycaena rhymnus *	
*Satyriumpruni* (Linnaeus, 1758)	* Papilio pruni *	
*Satyriumilicis* (Esper, 1779)	* Papilio ilicis *	
*Satyriumesculi* (Hübner, [1804])	* Papilio esculi *	
*Satyriumledereri* (Boisduval, 1848)	* Lycaena ledereri *	
*Satyriumw-album* (Knoch, 1782)	* Papilio w-album *	
*Satyriumspini* ([Denis & Schiffermüller], 1775)	* Papilio spini *	
*Satyriumacaciae* (Fabricius, 1787)	* Papilio acaciae *	
** Polyommatinae **		
*Leptotespirithous* (Linnaeus, 1767)	* Papilio pirithous *	
*Cyclyriuswebbianus* (Brullé, 1839)	* Polyommatus webbianus *	
*Azanusubaldus* (Stoll, 1782)	* Papilio ubaldus *	
*Azanusjesous* (Guérin-Méneville, 1849)	* Polyommatus jesous *	
*Lampidesboeticus* (Linnaeus, 1767)	* Papilio boeticus *	
*Cacyreusmarshalli* Butler, 1898	* Cacyreus marshalli *	
*Celastrinaargiolus* (Linnaeus, 1758)	* Papilio argiolus *	
*Tarucustheophrastus* (Fabricius, 1793)	* Hesperia theophrastus *	
*Tarucusbalkanicus* (Freyer, 1844)	* Lycaena balkanica *	5
*Phengarisalcon* ([Denis & Schiffermüller], 1775)	* Papilio alcon *	
*Phengarisarion* (Linnaeus, 1758)	* Papilio arion *	
*Phengaristeleius* (Bergsträsser, 1779)	* Papilio teleius *	
*Phengarisnausithous* (Bergsträsser, 1779)	* Papilio nausithous *	
*Turananataygetica* (Rebel, 1902)	* Lycaena panagaea taygetica *	
*Pseudophilotesbavius* (Eversmann, 1832)	* Lycaena bavius *	
*Pseudophilotesbarbagiae* De Prins & van der Poorten, 1982	* Pseudophilotes barbagiae *	
*Pseudophilotesabencerragus* (Pierret, 1837)	* Argus abencerragus *	
*Pseudophilotespanoptes* (Hübner, [1813])	* Papilio panoptes *	
*Pseudophilotesvicrama* (Moore, 1865)	* Polyommatus vicrama *	
*Pseudophilotesbaton* (Bergsträsser, 1779)	* Papilio baton *	
*Scolitantidesorion* (Pallas, 1771)	* Papilio orion *	
*Praephilotesanthracias* (Christoph, 1877)	* Lycaena anthracias *	
*Iolanaiolas* (Ochsenheimer, 1816)	* Lycaena iolas *	
*Iolanadebilitata* (Schultz, 1905)	Lycaena jolas var. debilitata	16
*Glaucopsychemelanops* (Boisduval, 1828)	* Polyommatus melanops *	
*Glaucopsychepaphos* Chapman, 1920	* Glaucopsyche paphos *	
*Glaucopsychealexis* (Poda, 1761)	* Papilio alexis *	
*Zizeeriaknysna* (Trimen, 1862)	* Lycaena knysna *	
*Zizeeriakarsandra* (Moore, 1865)	* Polyommatus karsandra *	
*Tongeiafischeri* (Eversmann, 1843)	* Lycaena fischeri *	
*Cupidoargiades* (Pallas, 1771)	* Papilio argiades *	
*Cupidodecoloratus* (Staudinger, 1886)	* Lycaena argiades decolorata *	5
*Cupidoalcetas* (Hoffmansegg, 1804)	* Papilio alcetas *	
*Cupidoosiris* (Meigen, 1829)	* Polyommatus osiris *	
*Cupidominimus* (Fuessly, 1775)	* Papilio minimus *	
*Cupidolorquinii* (Herrich-Schäffer, 1850)	* Lycaena lorquinii *	17
*Luthrodesgalba* (Lederer, 1855)	* Lycaena galba *	18
*Freyeriatrochylus* (Freyer, 1844)	* Lycaena trochylus *	18,19
*Plebejusargus* (Linnaeus, 1758)	* Papilio argus *	
*Plebejusidas* (Linnaeus, [1760])	* Papilio idas *	10
*Plebejusbellieri* (Oberthür, 1910)	* Lycaena bellieri *	
*Plebejusargyrognomon* (Bergsträsser, 1779)	* Papilio argyrognomon *	
*Agriadesorbitulus* (Prunner, 1798)	* Papilio orbitulus *	18
*Agriadesoptilete* (Knoch, 1781)	* Papilio optilete *	18
*Agriadespyrenaicus* (Boisduval, 1840)	Lycaena orbitulus var. pyrenaica	5, 18
*Agriadesdardanus* (Freyer, 1843)	* Lycaena dardanus *	18
*Agriadeszullichi* Hemming, 1933	* Agriades zullichi *	18
*Agriadesglandon* (Prunner, 1798)	* Papilio glandon *	18
*Agriadesaquilo* (Boisduval, 1832)	* Argus aquilo *	18
*Plebejidealoewii* (Zeller, 1847)	* Lycaena loewii *	18
*Eumedoniaeumedon* (Esper, 1780)	* Papilio eumedon *	18
*Kretaniapsylorita* (Freyer, 1845)	* Lycaena psylorita *	18
*Kretaniahesperica* (Rambur, 1839)	* Polyommatus hespericus *	5, 18
*Kretaniaeurypilus* (Freyer, 1851)	* Lycaena eurypilus *	18
*Kretaniatrappi* (Verity, 1927)	* Lycaena trappi *	18
*Kretaniasephirus* (Frivaldszky, 1835)	* Lycaena sephirus *	18
*Kretaniapylaon* (Fischer, 1832)	* Lycaena pylaon *	18
*Cyanirissemiargus* (Rottemburg, 1775)	* Papilio semiargus *	
*Glabroculuscyane* (Eversmann, 1837)	* Lycaena cyane *	18
*Ariciamorronensis* (Ribbe, 1910)	* Lycaena idas morronensis *	
*Ariciaanteros* (Freyer, 1838)	* Lycaena anteros *	
*Ariciacramera* (Eschscholtz, 1821)	* Lycaena cramera *	
*Aricianicias* (Meigen, 1829)	* Polyommatus nicias *	20
*Ariciaartaxerxes* (Fabricius, 1793)	* Hesperia artaxerxes *	
*Ariciamontensis* Verity, 1928	* Aricia medon montensis *	
*Ariciaagestis* ([Denis & Schiffermüller], 1775)	* Papilio agestis *	
*Neolysandracoelestina* (Eversmann, 1843)	* Lycaena coelestina *	18
*Lysandrahispana* (Herrich-Schäffer, 1851)	Lycaena coridon var. hispana	18
*Lysandracorydonius* (Herrich-Schäffer, 1852)	* Lycaena coridon corydonius *	18
*Lysandrabellargus* (Rottemburg, 1775)	* Papilio bellargus *	18
*Lysandracoridon* (Poda, 1761)	* Papilio coridon *	18
*Lysandracaelestissima* (Verity, 1921)	* Agriades coridon caelestissima *	18
*Lysandraalbicans* (Gerhard, 1851)	Lycaena coridon var. albicans	18
*Polyommatusescheri* (Hübner, [1823])	* Papilio escheri *	
*Polyommatusthersites* (Cantener, 1835)	* Argus thersites *	
*Polyommatusdaphnis* ([Denis & Schiffermüller], 1775)	* Papilio daphnis *	
*Polyommatusamandus* (Schneider, 1792)	* Papilio amandus *	
*Polyommatusgolgus* (Hübner, [1813])	* Papilio golgus *	
*Polyommatusnivescens* (Keferstein, 1851)	Lycaena dorylas var. nivescens	
*Polyommatusdorylas* ([Denis & Schiffermüller], 1775)	* Papilio dorylas *	
*Polyommatuscelina* (Austaut, 1879)	* Lycaena celina *	21
*Polyommatusicarus* (Rottemburg, 1775)	* Papilio icarus *	
*Polyommatuseros* (Ochsenheimer, 1808)	* Papilio eros *	
*Polyommatusdamon* ([Denis & Schiffermüller], 1775)	* Papilio damon *	
*Polyommatusdamone* (Eversmann, 1841)	* Lycaena damone *	
*Polyommatusdamocles* (Herrich-Schäffer, 1844)	* Lycaena damocles *	
*Polyommatusadmetus* (Esper, 1783)	* Papilio admetus *	
*Polyommatusripartii* (Freyer, 1830)	* Lycaena ripartii *	
*Polyommatusnephohiptamenos* (Brown & Coutsis, 1978)	* Agrodiaetus nephohiptamenos *	
*Polyommatusiphigenia* (Herrich-Schäffer, 1847)	* Lycaena iphigenia *	
*Polyommatusvioletae* (Gómez-Bustillo, Expósito & Martínez, 1979)	* Agrodiaetus violetae *	
*Polyommatusfulgens* (Sagarra, 1925)	Hirsutina dolus r. fulgens	22
*Polyommatusfabressei* (Oberthür, 1910)	Lycaena rippertii r. fabressei	
*Polyommatusdolus* (Hübner, [1823])	* Papilio dolus *	
*Polyommatushumedasae* (Toso & Balletto, 1976)	* Agrodiaetus humedasae *	
*Polyommatustimfristos* Lukhtanov, Vishnevskaya & Shapoval, 2016	* Polyommatus timfristos *	23
*Polyommatusorphicus* Kolev, 2005	* Polyommatus orphicus *	
*Polyommatusaroaniensis* (Brown, 1976)	* Agrodiaetus alcestis aroaniensis *	
** Nymphalidae **		
** Limenitidinae **		
*Neptissappho* (Pallas, 1771)	* Papilio sappho *	
*Neptisrivularis* (Scopoli, 1763)	* Papilio rivularis *	
*Limenitisreducta* Staudinger, 1901	* Limenitis camilla reducta *	
*Limenitispopuli* (Linnaeus, 1758)	* Papilio populi *	
*Limenitiscamilla* (Linnaeus, 1764)	* Papilio camilla *	
** Heliconiinae **		
*Issorialathonia* (Linnaeus, 1758)	* Papilio lathonia *	
*Issoriaeugenia* (Eversmann, 1847)	* Argynnis eugenia *	
*Brenthishecate* ([Denis & Schiffermüller], 1775)	* Papilio hecate *	
*Brenthisino* (Rottemburg, 1775)	* Papilio ino *	
*Brenthisdaphne* ([Denis & Schiffermüller], 1775)	* Papilio daphne *	
*Argynnispaphia* (Linnaeus, 1758)	* Papilio paphia *	
*Argynnispandora* ([Denis & Schiffermüller], 1775)	* Papilio pandora *	
*Argynnislaodice* (Pallas, 1771)	* Papilio laodice *	
*Speyeriaaglaja* (Linnaeus, 1758)	* Papilio aglaja *	24
*Fabricianaelisa* (Godart, 1823)	* Argynnis elisa *	24
*Fabriciananiobe* (Linnaeus, 1758)	* Papilio niobe *	24
*Fabricianaadippe* ([Denis & Schiffermüller], 1775)	* Papilio adippe *	24
*Boloriaeunomia* (Esper, 1800)	* Papilio eunomia *	25
*Boloriagraeca* (Staudinger, 1870)	* Argynnis pales graeca *	
*Boloriapales* ([Denis & Schiffermüller], 1775)	* Papilio pales *	
*Boloriaalaskensis* (Holland, 1900)	* Argynnis alaskensis *	
*Bolorianapaea* (Hoffmansegg, 1804)	* Papilio napaea *	
*Boloriaaquilonaris* (Stichel, 1908)	* Argynnis aquilonaris *	
*Boloriatritonia* (Böber, 1812)	* Papilio tritonia *	
*Boloriapolaris* (Boisduval, 1828)	* Argynnis polaris *	
*Boloriathore* (Hübner, [1804])	* Papilio thore *	26
*Boloriaselene* ([Denis & Schiffermüller], 1775)	* Papilio selene *	
*Boloriaeuphrosyne* (Linnaeus, 1758)	* Papilio euphrosyne *	
*Boloriadia* (Linnaeus, 1767)	* Papilio dia *	
*Boloriaimproba* (Butler, 1877)	* Argynnis improba *	
*Boloriafrigga* (Thunberg, 1791)	* Papilio frigga *	27
*Boloriafreija* (Thunberg, 1791)	* Papilio freija *	27
*Boloriaselenis* (Eversmann, 1837)	* Argynnis selenis *	
*Boloriaoscarus* (Eversmann, 1844)	* Argynnis oscarus *	
*Boloriatitania* (Esper, [1793])	* Papilio titania *	
*Boloriachariclea* (Schneider, 1794)	* Papilio chariclea *	
*Boloriaangarensis* (Erschoff, 1870)	* Argynnis angarensis *	
** Apaturinae **		
*Apaturairis* (Linnaeus, 1758)	* Papilio iris *	
*Apaturametis* Freyer, 1829	* Apatura metis *	
*Apaturailia* ([Denis & Schiffermüller], 1775)	* Papilio ilia *	
** Nymphalinae **		
*Araschnialevana* (Linnaeus, 1758)	* Papilio levana *	
*Vanessavirginiensis* (Drury, 1773)	* Papilio cardui virginiensis *	
*Vanessacardui* (Linnaeus, 1758)	* Papilio cardui *	
*Vanessavulcania* Godart, 1819	* Vanessa vulcania *	
*Vanessaatalanta* (Linnaeus, 1758)	* Papilio atalanta *	
*Aglaisio* (Linnaeus, 1758)	* Papilio io *	
*Aglaisurticae* (Linnaeus, 1758)	* Papilio urticae *	
*Aglaisichnusa* (Hübner, [1824])	* Papilio ichnusa *	28
*Polygoniaegea* (Cramer, 1775)	* Papilio egea *	
*Polygoniac-album* (Linnaeus, 1758)	* Papilio c-album *	
*Nymphalisvaualbum* ([Denis & Schiffermüller], 1775)	* Papilio vau album *	
*Nymphalispolychloros* (Linnaeus, 1758)	* Papilio polychloros *	
*Nymphalisxanthomelas* ([Denis & Schiffermüller], 1775)	* Papilio xanthomelas *	
*Nymphalisantiopa* (Linnaeus, 1758)	* Papilio antiopa *	
*Hypolimnasmisippus* (Linnaeus, 1764)	* Papilio misippus *	
*Euphydryasdesfontainii* (Godart, 1819)	* Papilio desfontainii *	
*Euphydryasaurinia* (Rottemburg, 1775)	* Papilio aurinia *	
*Euphydryascynthia* ([Denis & Schiffermüller], 1775)	* Papilio cynthia *	
*Euphydryasiduna* (Dalman, 1816)	* Melitaea iduna *	
*Euphydryasmaturna* (Linnaeus, 1758)	* Papilio maturna *	
*Euphydryasintermedia* (Ménétriés, 1859)	* Melitaea maturna intermedia *	
*Melitaeatrivia* ([Denis & Schiffermüller], 1775)	* Papilio trivia *	
*Melitaeadidyma* (Esper, 1778)	* Papilio didyma *	
*Melitaeaarduinna* (Esper, 1783)	* Papilio arduinna *	
*Melitaeaaetherie* (Hübner, [1826])	* Papilio aetherie *	
*Melitaeaphoebe* ([Denis & Schiffermüller], 1775)	* Papilio phoebe *	
*Melitaeaornata* Christoph, 1893	* Melitaea phoebe ornata *	29
*Melitaeacinxia* (Linnaeus, 1758)	* Papilio cinxia *	
*Melitaeadiamina* (Lang, 1789)	* Papilio diamina *	
*Melitaeaceladussa* Fruhstorfer, 1910	* Melitaea athalia celadussa *	30
*Melitaeadeione* (Geyer, [1832])	* Papilio deione *	
*Melitaeabritomartis* Assmann, 1847	* Melitaea britomartis *	
*Melitaeaathalia* (Rottemburg, 1775)	* Papilio athalia *	
*Melitaeavaria* Herrich-Schäffer, 1851	* Melitaea varia *	31
*Melitaeaparthenoides* Keferstein, 1851	* Melitaea athalia parthenoides *	
*Melitaeaaurelia* Nickerl, 1850	* Melitaea aurelia *	
*Melitaeaasteria* Freyer, 1828	* Melitaea asteria *	
** Libytheinae **		
*Libytheaceltis* (Laicharting, 1782)	* Papilio celtis *	
** Danainae **		
*Danausplexippus* (Linnaeus, 1758)	* Papilio plexippus *	
*Danauschrysippus* (Linnaeus, 1758)	* Papilio chrysippus *	
** Charaxinae **		
*Charaxesjasius* (Linnaeus, 1767)	* Papilio jasius *	
** Satyrinae **		
*Coenonymphaphryne* (Pallas, 1771)	* Papilio phryne *	
*Coenonymphaoedippus* (Fabricius, 1787)	* Papilio oedippus *	
*Coenonymphadorus* (Esper, 1782)	* Papilio dorus *	
*Coenonymphathyrsis* (Freyer, 1845)	* Hipparchia thyrsis *	
*Coenonymphapamphilus* (Linnaeus, 1758)	* Papilio pamphilus *	
*Coenonymphatullia* (Müller, 1764)	* Papilio tullia *	
*Coenonympharhodopensis* Elwes, 1900	* Coenonympha tiphon rhodopensis *	
*Coenonymphaamaryllis* (Stoll, 1782)	* Papilio amaryllis *	
*Coenonymphaglycerion* (Borkhausen, 1788)	* Papilio glycerion *	
*Coenonymphacorinna* (Hübner, [1804])	* Papilio corinna *	
*Coenonymphaleander* (Esper, 1784)	* Papilio leander *	
*Coenonymphahero* (Linnaeus, [1760])	* Papilio hero *	10
*Coenonymphagardetta* (Prunner, 1798)	* Papilio gardetta *	
*Coenonymphaorientalis* Rebel, 1909	Coenonympha arcania var. orientalis	32
*Coenonymphaarcania* (Linnaeus, [1760])	* Papilio arcania *	10
*Kiriniaroxelana* (Cramer, 1777)	* Papilio roxelana *	
*Kiriniaclimene* (Esper, 1783)	* Papilio climene *	
*Lopingaachine* (Scopoli, 1763)	* Papilio achine *	
*Parargexiphia* (Fabricius, 1775)	* Papilio xiphia *	
*Parargexiphioides* Staudinger, 1871	* Pararge xiphia xiphioides *	
*Parargeaegeria* (Linnaeus, 1758)	* Papilio aegeria *	
*Lasiommatamaera* (Linnaeus, 1758)	* Papilio maera *	
*Lasiommatadeidamia* (Eversmann, 1851)	* Hipparchia deidamia *	
*Lasiommatapetropolitana* (Fabricius, 1787)	* Papilio maera petropolitana *	
*Lasiommataparamegaera* (Hübner, [1824])	* Papilio paramegaera *	
*Lasiommatamegera* (Linnaeus, 1767)	* Papilio megera *	
*Melanargiarussiae* (Esper, 1783)	* Papilio russiae *	
*Melanargialarissa* (Geyer, [1828])	* Papilio larissa *	
*Melanargialachesis* (Hübner, 1790)	* Papilio lachesis *	
*Melanargiagalathea* (Linnaeus, 1758)	* Papilio galathea *	
*Melanargiaines* (Hoffmansegg, 1804)	* Papilio ines *	
*Melanargiaarge* (Sulzer, 1776)	* Papilio arge *	
*Melanargiapherusa* (Boisduval, 1833)	* Arge pherusa *	
*Melanargiaoccitanica* (Esper, [1793])	* Papilio arge occitanica *	
*Hipparchiafatua* Freyer, 1843	* Hipparchia fatua *	33
*Hipparchiastatilinus* (Hufnagel, 1766)	* Papilio statilinus *	
*Hipparchiatilosi* Manil, 1984	* Hipparchia wyssii tilosi *	
*Hipparchiabacchus* (Higgins, 1967)	* Pseudotergumia wyssii bacchus *	
*Hipparchiawyssii* (Christ, 1889)	* Satyrus fidia wyssii *	
*Hipparchiatamadabae* Owen & Smith, 1992	* Hipparchia wyssi tamadabae *	
*Hipparchiagomera* (Higgins, 1967)	* Pseudotergumia wyssii gomera *	
*Hipparchiafidia* (Linnaeus, 1767)	* Papilio fidia *	
*Hipparchianeomiris* (Godart, 1823)	* Satyrus neomiris *	34
*Hipparchiaautonoe* (Esper, 1783)	* Papilio autonoe *	
*Hipparchiahermione* (Linnaeus, 1764)	* Papilio hermione *	
*Hipparchiasyriaca* (Staudinger, 1871)	* Satyrus hermione syriaca *	
*Hipparchiafagi* (Scopoli, 1763)	* Papilio fagi *	
*Hipparchiamersina* (Staudinger, 1871)	* Satyrus semele mersina *	
*Hipparchiamiguelensis* (Le Cerf, 1935)	* Satyrus azorinus miguelensis *	
*Hipparchiaazorina* (Strecker, 1899)	* Satyrus azorinus *	5, 35
*Hipparchiasenthes* (Fruhstorfer, 1908)	* Eumenis semele senthes *	
*Hipparchiamaderensis* (Bethune-Baker, 1891)	* Satyrus semele maderensis *	
*Hipparchiasemele* (Linnaeus, 1758)	* Papilio semele *	
*Hipparchiablachieri* (Fruhstorfer, 1908)	* Eumenis semele blachieri *	
*Hipparchiaaristaeus* (Bonelli, 1826)	* Papilio aristaeus *	
*Hipparchiavolgensis* (Mazokhin-Porshnyakov, 1952)	* Satyrus semele volgensis *	
*Hipparchianeapolitana* (Stauder, 1921)	* Satyrus neapolitana *	
*Hipparchialeighebi* Kudrna, 1976	* Hipparchia semele leighebi *	
*Hipparchiapellucida* (Stauder, 1924)	* Satyrus semele pellucida *	36
*Hipparchiasbordonii* Kudrna, 1984	* Hipparchia sbordonii *	
*Hipparchiacypriensis* (Holik, 1949)	* Satyrus semele cypriensis *	
*Hipparchiacretica* (Rebel, 1916)	* Satyrus semele cretica *	
*Hipparchiachristenseni* Kudrna, 1977	* Hipparchia christenseni *	
*Minoisdryas* (Scopoli, 1763)	* Papilio dryas *	
*Brintesiacirce* (Fabricius, 1775)	* Papilio circe *	
*Arethusanaarethusa* ([Denis & Schiffermüller], 1775)	* Papilio arethusa *	
*Oeneistarpeia* (Pallas, 1771)	* Papilio tarpeia *	
*Oeneisbore* (Schneider, 1792)	* Papilio bore *	
*Oeneisammon* Elwes, 1899	Oeneis bore var. ammon	37
*Oeneismelissa* (Fabricius, 1775)	* Papilio melissa *	
*Oeneismagna* Graeser, 1888	* Oeneis jutta magna *	
*Oeneisjutta* (Hübner, [1806])	* Papilio jutta *	
*Oeneisnorna* (Thunberg, 1791)	* Papilio norna *	
*Oeneispolixenes* (Fabricius, 1775)	* Papilio polixenes *	
*Oeneisglacialis* (Moll, 1785)	* Papilio glacialis *	38
*Satyrusferula* (Fabricius, 1793)	* Papilio ferula *	
*Satyrusvirbius* Herrich-Schäffer, 1844	* Satyrus virbius *	
*Satyrusactaea* (Esper, 1781)	* Papilio actaea *	
*Chazarabriseis* (Linnaeus, 1764)	* Papilio briseis *	
*Chazaraprieuri* (Pierret, 1837)	* Satyrus prieuri *	
*Chazarapersephone* (Hübner, [1805])	* Papilio persephone *	
*Pseudochazarageyeri* (Herrich-Schäffer, 1846)	* Satyrus geyeri *	
*Pseudochazaragraeca* (Staudinger, 1870)	* Satyrus pelopea graeca *	
*Pseudochazaraamymone* Brown, 1976	* Pseudochazara amymone *	
*Pseudochazaraanthelea* (Hübner, [1824])	* Papilio anthelea *	
*Pseudochazaraamalthea* (Frivaldszky, 1845)	* Hipparchia amalthea *	39
*Pseudochazarawilliamsi* (Romei, 1927)	* Satyrus hippolyte williamsi *	
*Pseudochazaraeuxina* (Kuznetsov, 1909)	* Hipparchia euxina *	
*Pseudochazaramercurius* (Staudinger, 1887)	* Satyrus mercurius *	40
*Pseudochazaracingovskii* (Gross, 1973)	* Satyrus sintenisi cingovskii *	
*Pseudochazaratisiphone* Brown, [1981]	* Pseudochazara cingovskii tisiphone *	39
*Pseudochazaraorestes* De Prins & van der Poorten, 1981	* Pseudochazara orestes *	
*Ypthimaasterope* (Klug, 1832)	* Hipparchia asterope *	
*Proterebiaphegea* (Borkhausen, 1788)	* Papilio phegea *	41
*Hyponephelehuebneri* Koçak, 1980	* Hyponephele huebneri *	
*Hyponephelelycaon* (Kühn, 1774)	* Papilio lycaon *	
*Hyponephelelupina* (Costa, 1836)	* Satyrus lupinus *	5
*Aphantopushyperantus* (Linnaeus, 1758)	* Papilio hyperantus *	
*Pyroniacecilia* (Vallantin, 1894)	* Epinephele ida cecilia *	
*Pyroniatithonus* (Linnaeus, 1771)	* Papilio tithonus *	42
*Pyroniabathseba* (Fabricius, 1793)	* Papilio bathseba *	
*Maniolajurtina* (Linnaeus, 1758)	* Papilio jurtina *	
*Maniolanurag* (Ghiliani, 1852)	* Satyrus nurag *	
*Maniolachia* Thomson, 1987	* Maniola chia *	
*Maniolamegala* (Oberthür, 1909)	* Epinephele janira megala *	
*Maniolacypricola* (Graves, 1928)	* Epinephele cypricola *	
*Maniolatelmessia* (Zeller, 1847)	* Hipparchia telmessia *	
*Maniolahalicarnassus* Thomson, 1990	* Maniola halicarnassus *	
*Erebiaedda* Ménétriés, 1851	* Erebia edda *	
*Erebiafasciata* Butler, 1868	* Erebia fasciata *	
*Erebiadiscoidalis* (Kirby, 1837)	* Hipparchia discoidalis *	
*Erebiarossii* (Curtis, 1835)	* Hipparchia rossii *	43
*Erebiacyclopius* (Eversmann, 1844)	* Hipparchia cyclopius *	
*Erebiaembla* (Thunberg, 1791)	* Papilio embla *	
*Erebiadisa* (Thunberg, 1791)	* Papilio disa *	
*Erebiameolans* (Prunner, 1798)	* Papilio meolans *	
*Erebiadabanensis* Erschoff, 1872	* Erebia dabanensis *	44
*Erebiajeniseiensis* Trybom, 1877	* Erebia ligea jeniseiensis *	
*Erebiaclaudina* (Borkhausen, 1789)	* Papilio claudina *	
*Erebiamanto* ([Denis & Schiffermüller], 1775)	* Papilio manto *	
*Erebiaottomana* Herrich-Schäffer, 1847	* Erebia dromus ottomana *	
*Erebiahispania* Butler, 1868	* Erebia hispania *	
*Erebiarondoui* Oberthür, 1908	* Erebia rondoui *	
*Erebiacallias* Edwards, 1871	* Erebia callias *	45
*Erebiatyndarus* (Esper, 1781)	* Papilio tyndarus *	
*Erebiacassioides* (Hohenwarth, 1792)	* Papilio cassioides *	46
*Erebianivalis* Lorković & Lesse, 1954	* Erebia nivalis *	
*Erebianeleus* (Freyer, 1832)	* Hipparchia neleus *	47
*Erebiacalcarius* Lorković, 1953	* Erebia tyndarus calcarius *	
*Erebiaarvernensis* Oberthür, 1908	* Erebia tyndarus arvernensis *	47
*Erebiaoeme* (Hübner, [1804])	* Papilio oeme *	
*Erebiagorge* (Hübner, [1804])	* Papilio gorge *	
*Erebiasthennyo* Graslin, 1850	* Erebia sthennyo *	
*Erebiapandrose* (Borkhausen, 1788)	* Papilio pandrose *	
*Erebiaeriphyle* (Freyer, 1836)	* Hipparchia eriphyle *	
*Erebiaepistygne* (Hübner, [1819])	* Papilio epistygne *	
*Erebiaeuryale* (Esper, 1805)	* Papilio euryale *	
*Erebiapalarica* Chapman, 1905	* Erebia palarica *	
*Erebialigea* (Linnaeus, 1758)	* Papilio ligea *	
*Erebiapluto* (Prunner, 1798)	* Papilio pluto *	
*Erebiaaethiopellus* (Hoffmansegg, 1806)	* Papilio aethiopellus *	
*Erebiagorgone* Boisduval, 1833	* Erebia gorgone *	
*Erebiarhodopensis* Nicholl, 1900	* Erebia gorgone rhodopensis *	
*Erebiamnestra* (Hübner, [1804])	* Papilio mnestra *	
*Erebiaalbergana* (Prunner, 1798)	* Papilio alberganus *	5
*Erebiasudetica* Staudinger, 1861	* Erebia melampus sudetica *	
*Erebiamelampus* (Fuessly, 1775)	* Papilio melampus *	
*Erebiatriarius* (Prunner, 1798)	* Papilio triarius *	
*Erebiapolaris* Staudinger, 1861	Erebia medusa var. polaris	48
*Erebiamedusa* ([Denis & Schiffermüller], 1775)	* Papilio medusa *	
*Erebiaaethiops* (Esper, 1777)	* Papilio aethiops *	
*Erebiapharte* (Hübner, [1804])	* Papilio pharte *	
*Erebiachristi* Rätzer, 1890	* Erebia christi *	
*Erebiaorientalis* Elwes, 1900	* Erebia epiphron orientalis *	
*Erebiaepiphron* (Knoch, 1783)	* Papilio epiphron *	
*Erebiaflavofasciata* Heyne, 1895	* Erebia flavofasciata *	
*Erebiamontana* (Prunner, 1798)	* Papilio montanus *	5
*Erebiastyx* (Freyer, 1834)	* Hipparchia styx *	
*Erebiastiria* (Godart, [1824])	* Satyrus stirius *	5
*Erebiascipio* Boisduval, 1833	* Erebia scipio *	49
*Erebiapronoe* (Esper, 1780)	* Papilio pronoe *	
*Erebiamelas* (Herbst, 1796)	* Papilio melas *	
*Erebialefebvrei* (Boisduval, 1828)	* Satyrus lefebvrei *	
*Erebiazapateri* Oberthür, 1875	* Erebia zapateri *	
*Erebianeoridas* (Boisduval, 1828)	* Satyrus neoridas *	

**Table 3.** Annotations to the updated checklist of the butterflies of Europe.

**1**	*Iphiclidesfeisthamelii* is considered a separate species based on differences in adult morphology (Coutsis and van Oorschot 2011, Lafranchis et al. 2015) and nuclear genetic markers (Wiemers and Gottsberger 2010; Dincă et al. 2015), despite very local hybridisation along the contact zone in southern France (Lafranchis et al. 2015) and extensive mitochondrial introgression in the Iberian Peninsula (Wiemers and Gottsberger 2010; Dincă et al. 2015). Its distribution includes the SW part of France, the Iberian Peninsula, and northern Africa.
**2**	Author of the name is Giuseppe Gené (1800–1847), not Achille Guenée.
**3**	Dapporto (2009) has shown that *Zerynthiacassandra* from peninsular Italy is a separate species based on differences in genital morphology. This was further confirmed by molecular studies (Zinetti et al. 2013).
**4**	*Spialiarosae* has been recognised as a separate species endemic to mountains of Spain based on differences in ecology and evidence from molecular studies (mitochondrial DNA, chemical profiles) (Hernández-Roldán et al. 2016, 2018). The species has already been included in Fauna Europaea (2018).
**5**	Gender agreement changes were applied consistently in accordance with Art. 31.2 and Art. 34.2 (ICZN 1999).
**6**	As descriptions of both *Syrichtusalveus f. foulquieri* and *Syrichtusalveus f. bellieri* were published simultaneously (Oberthür, 1910), the name used by the first reviser (i. e. Rebel 1914), *Pyrgusfoulquieri*, should be used in accordance with Art. 24.2.1 and Art. 24.2.2 (ICZN 1999).
**7**	Recent studies have shown that *Leptideareali* actually comprises two species, *L.reali* and *L.juvernica*. *L.reali* is known from south-western Europe (Spain, S France and Italy) and is replaced by *L.juvernica* in the rest of the continent (Dincă et al. 2011b). *L.sinapis*, *L.reali*, and *L.juvernica* are reproductively isolated due to female mate choice (Dincă et al. 2013).
**8**	The year of the publication of the name *Anteoscleobule* is 1831, not 1830 (the original plate [79], published in 1824, carried no names).
**9**	The name *Papiliocroceus* should be credited to Geoffroy in Fourcroy, 1785, not to Fourcroy (Ganglbauer and Heyden 1906, D'Aguilar and Raimbault 1990, Grieshuber et al. 2012).
**10**	The date of the publication of the names by Linnaeus in Fauna Svecica (ed. 2) is 14 November 1760, not 1761 (see Evenhuis 1997, Bousquet 2016).
**11**	The year of the publication of the name *Pierisbalcana* is 1969, not 1970. The publication year of volume 21 (1–4) (1968) of *Biološki glasnik* [= volume 70 of *Periodicum Biologorum*] is printed on the cover page as “1969” and, moreover, Lorković´s personal copy held in the Croatian Natural History museum has a hand written addition of the publication year “1969” in the header of his article (Šašić, pers. comm.). Additionally, the author´s name is misspelled and should be Lorković (see also Lorković 1969).
**12**	According to Lvovsky and Morgun (2007) the species is present in Russia south of the Urals in the Orenburg region. The subspecies *Lycaenadimorphairghiza* was originally described as a subspecies of *L.japhetica* (Nekrutenko 1985), but we follow the decision in the taxonomic review by Lukhtanov (2000).
**13**	The year of the publication of the name *Polyommatusottomanus* is 1831, not 1830. Lefèbvre cited the date 1830, which corresponds to the date of submission of his article, but the issue of the journal was published in January 1831. See Lefèbvre (1831)
**14**	The generic names *Apharitis* and *Spindasis* were synonymised with *Cigaritis* due to morphological similarities (see Heath and Pringle 2011).
**15**	The name *Papilioroboris* was first published in 1793, not 1789 (Lamas 2013).
**16**	*Iolanadebilitata* has been recognised as a separate species based on constant differences in adult morphology (Dumont 2004) and mitochondrial DNA – barcoding gene (Dincă et al. 2015).
**17**	The year of the publication of the name and plates for *Cupidolorquinii* is 1850, not 1847 (Hemming 1937, Heppner 1982).
**18**	Genus level classiﬁcation in the subfamily Polyommatinae follows Talavera et al. (2013) based on molecular phylogeny. This arrangement partially concurs with differences in genital morphology (see Balletto et al. 2014, Coutsis 2017).
**19**	The year of the publication of the name *Lycaenatrochylus* is 1844, not 1845 (Tremewan 1988, Olivier 2000).
**20**	The year of the publication of the name *Polyommatusnicias* is ante September 1829, not 1830 (Griffin 1931).
**21**	*Polyommatuscelina* has been recognised as a separate species distributed in the Iberian Peninsula, northern Africa, Sardinia and Sicily based on molecular markers and adult morphology (Wiemers et al. 2010; Dincă et al. 2011a).
**22**	The author´s surname Sagarra should be without the particle “de”. It is listed as such in the members list of the Institució Catalana d'Història Natural in 1925 bulletin Vol. 5 – Num. 1. Generally, when the particle is written in lowercase, it should be treated as a suffix that goes after the first name (Welter-Schultes 2013).
**23**	*Polyommatustimfristos* is considered a separate species due to differences in haploid chromosome number compared to *P.aroaniensis* and mitochondrial DNA – barcoding gene (Vishnevskaya et al. 2016).
**24**	Genus level classiﬁcation in the tribe Argynnini follows De Moya et al. (2017) based on molecular phylogenetics. It is corroborated by extensive differences in genital morphology (Simonsen 2006a, 2006b).
**25**	The name *Papilioeunomia* was first published in 1800, not 1799 (Poche, 1938).
**26**	The name *Papiliothore* was first published in 1804, not 1803 (Hemming 1937).
**27**	Description of *Boloriafreija* and *Boloriafrigga* must be credited to Thunberg, not to Becklin (Thunberg wrote Becklin's dissertation), see Karsholt and Nielsen (1986).
**28**	*Papilioichnusa* was first described by Hübner (ante 23 December) 1824. *Vanessaichnusa* Bonelli was published in February 1825 and is a junior secondary homonym and junior subjective synonym, see Hemming (1937).
**29**	Among the species with red headed larvae within the *Melitaeaphoebe* species group only *M.ornata* is present in Europe in southeastern Russia, the Balkan Peninsula, Spain, southeastern France, and southern Italy. *M.telona* is limited to the Levant and *M.punica* to northern Africa (Toth et al. 2014).
**30**	*Melitaeaceladussa* Fruhstorfer, 1910 is considered a separate species distributed in western Europe that differs in genital morphology (Higgins 1932) and molecular markers (Leneveu et al. 2009, Dincă et al. 2015) from *M.athalia*, with hybrids known from the contact zone (Achtelik 2006; Oorschot and Coutsis 2014). The species was referred to also as *M.nevadensis* Oberthür, 1904, which is a junior primary homonym of Melitaeaparthenievar.nevadensis Spuler, 1901, currently regarded as a junior subjective synonym of *Melitaeaparthenoides* Keferstein, 1851.
**31**	*Melitaeavaria* was first described by Herrich-Schäffer (1851) in *Systematische Bearbeitung der Schmetterlinge von Europa* Vol. 6(48): 2 (Hemming 1937). Melitaeaparthenievar.varia Meyer-Dür, 1852 (not 1851) is a junior primary homonym.
**32**	The name *Coenonympha arcánia* var. *orientális* [sic] appeared in part 4 of the ninth edition of Berge’s *Schmetterlingsbuch*, which was published on 22 May 1909 (Lempke 1949), not in 1910.
**33**	The name *Hipparchiafatua* was first published in 1843, not 1844 (Olivier 2000).
**34**	The name *Satyrusneomiris* was first published in 1823, not 1822. *Satyrusneomiris* first appeared on page 19 in Godart’s *Tableau méthodique des lépidoptères*..., published in 1823. The vernacular name Godart used in vol. 2 of *Hist. nat. Lépid. Pap. France*, pp. 88–89, pl. 11, figs. 1–2 (1822), »Satyre néomiris«, is unavailable, as it is not a scientific name.
**35**	The name *Satyrusazorinus* was first published in 1899, not 1898.
**36**	The name *Satyrussemelepellucida* was first published on 15 May 1924, not in 1923.
**37**	*Oeneisammon* is present in Europe in the Polar Urals (Tsvetkov 2006).
**38**	The name *Papilioglacialis* was first published in 1785, not 1783.
**39**	Based on differentiation in mtDNA (barcodes) and differences in morphology, *Pseudochazaraamalthea* and *P.tisiphone* are considered separate species from allopatric *P.anthelea* and *P.mniszechii* respectively (Verovnik and Wiemers 2016).
**40**	*Pseudochazarahippolyte* (Esper, 1783) is a junior primary homonym of *Papiliohyppolite* Drury, 1782. The oldest available name for this taxon is *Satyrusmercurius* Staudinger, 1887.
**41**	*Papilioafer* Esper, 1783 is a junior primary homonym of *Papilioafer* Drury, 1782 (see Koçak 1981), as is *Papilioafra* Fabricius, 1787, because it differs only in gender. Therefore the oldest available name is *Papiliophegea* Borkhausen, 1788.
**42**	The name *Papiliotithonus* was first published in 1771 in *Mantissa Plantarum Altera*, not in 1767.
**43**	The name *Hipparchiarossii* was first published in November 1835, not in 1834.
**44**	The name *Erebiadabanensis* was published on 13 November 1872, not in 1871.
**45**	Recently, a population of *Erebia* was discovered in the Polar Urals and described as a new species, *E.churkini* Bogdanov, 2008, but is now considered a subspecies of *Erebiacallias* (Tatarinov & Gorbunov, 2015). However, no further material is available, therefore it is tentatively considered as part of the European fauna. *Erebiacallias* is a member of the *tyndarus* group (Albre et al. 2008) and ranges from the mountains of the Asian part of Russia and Mongolia to Colorado (USA).
**46**	The author of the name *Papiliocassioides* is Hohenwarth alone as indicated on page III of Reiner and Hohenwarth (1792), not Reiner and Hohenwarth.
**47**	Based on molecular data and differences in wing patterns *Erebiacassioides* has been split into three allopatric species (Schmitt et al. 2016). *E.cassioides* is limited to the eastern Alps, *E.arvernensis* is distributed in the western Alps, Cantabrian mountains and Pyrénées, while *E.neleus* is present in the mountains of the Balkan Peninsula and the southern Carpathians.
**48**	The name *Erebiamedusapolaris* was first published in September 1861, not in 1871.
**49**	The year of publication of the name *Erebiascipio* by Boisduval is 1833, not 1832 (Cowan 1970).

**Table 4.** Species richness of European butterfly families and subfamilies.

Family	Subfamily	Genera	Species
** Hesperiidae **		**13**	**47**
	Hesperiinae	6	11
	Heteropterinae	2	3
	Pyrginae	5	33
** Lycaenidae **		**39**	**130**
	Aphnaeinae	1	1
	Lycaeninae	1	13
	Polyommatinae	30	98
	Theclinae	7	18
** Nymphalidae **		**41**	**246**
	Apaturinae	1	3
	Charaxinae	1	1
	Danainae	1	2
	Heliconiinae	6	32
	Libytheinae	1	1
	Limenitidinae	2	5
	Nymphalinae	8	37
	Satyrinae	21	165
** Papilionidae **		**5**	**15**
	Papilioninae	2	5
	Parnassiinae	3	10
** Pieridae **		**11**	**57**
	Coliadinae	3	18
	Dismorphiinae	1	5
	Pierinae	7	34
** Riodinidae **		**1**	**1**
	Nemeobiinae	1	1
**Total**	**21**	**110**	**496**

**Table 5.** Authors of currently valid European butterfly species (with a minimum of three described taxa).

**Author**	**Life data**	**Nationality**	**Species**	**Period**
Linnaeus, Carolus	1707–1778	Swedish	71	1758–1771
Poda von Neuhaus, Nicolaus (Nikolaus)	1723–1798	Austrian	4	1761
Scopoli, Giovanni Antonio	1723–1788	Italian	4	1763
Pallas, Peter Simon	1741–1811	German	8	1771
Schiffermüller, Johann Ignaz	1727–1806	Austrian	21	1775
Fabricius, Johan Christian	1745–1808	Danish	16	1775–1793
Rothenburg [alias Rottemburg], Siegmund Adrian von	1745–1797	German	8	1775
Esper, Eugen Johann Christoph	1742–1810	German	32	1777–1805
Bergsträsser, Johann Andreas Benignus	1732–1812	German	5	1779–1780
Knoch, August Wilhelm	1742–1818	German	3	1781–1783
Borkhausen, Moritz Balthasar	1760–1806	German	4	1788–1789
Hübner, Jacob	1761–1826	German	31	1790–1831
Thunberg, Carl Peter	1743–1828	Swedish	5	1791
Schneider, David Hinrich	1755–1826	German	3	1792–1794
Prunner, Leonhard von	17??–1830	German	8	1798
Hoffmansegg, Johann Centurius Graf von	1766–1849	German	6	1804–1806
Ochsenheimer, Ferdinand	1767–1822	German	4	1808–1816
Godart, Jean Baptiste	1775–1825	French	6	1819–1824
Freyer, Christian Friedrich	1794–1885	German	16	1828–1851
Boisduval, Jean Baptiste Alphonse Dechauffour de	1799–1879	French	13	1828–1848
Geyer, Carl	1802–1889	German	4	1828–1832
Klug, Johann Christoph Friedrich	1775–1856	German	4	1829–1834
Meigen, Johann Wilhelm	1764–1845	German	3	1829
Eversmann, Eduard Friedrich von	1794–1860	Russian	14	1832–1851
Rambur, Jules Pierre	1801–1870	French	10	1832–1839
Herrich-Schäffer, Gottlieb August Wilhelm	1799–1874	German	14	1844–1852
Zeller, Philipp Christoph	1808–1883	German	4	1847
Lederer, Julius	1821–1870	Austrian	3	1855–1864
Staudinger, Otto	1830–1900	German	17	1860–1901
Butler, Arthur Gardiner	1844–1925	British	6	1868–1898
Oberthür, Charles	1845–1924	French	9	1875–1910
Rebel, Hans	1861–1940	Austrian	5	1894–1916
Elwes, Henry John	1846–1922	British	3	1899–1900
Chapman, Thomas Algernon	1842–1921	British	3	1905–1920
Fruhstorfer, Hans	1866–1922	German	3	1908–1910
Verity, Ruggero	1883–1959	Italian	5	1921–1928
Kudrna, Otakar	1939–	Czech	3	1976–1984
Brown, John	19??–	British	3	1976–1981

**Table 6.** Butterfly species excluded from the European list with explanations.

* Turanana panagaea *	Distributed outside Europe in the Asian part of Turkey and replaced by *Turananataygetica* in Europe (Hesselbarth et al. 1995; Coutsis 2005). [Junior subjective synonym of *Lycaenaendymion* Gerhard, 1851; misspelled as *Turananapanagea* in Fauna Europaea]
(Herrich-Schäffer, 1851)	
* Polyommatus eleniae *	Considered conspecific with *Polyommatusorphicus* based on the equal haploid chromosome number and no differences in mitochondrial DNA – barcoding gene (Vishnevskaya et al. 2016).
Coutsis & De Prins, 2005	
* Polyommatus galloi *	According to the molecular study of Vila et al. (2010)*P.galloi* represents an isolated population of *Polyommatusripartii* and is not considered as a separate species.
(Balletto & Toso, 1979)	
* Polyommatus menalcas *	Distributed outside Europe in Asian part of Turkey (Hesselbarth et al. 1995).
(Freyer, 1837)	
* Polyommatus pljushtchi *	Species status is based on erroneous sequences (opinion in Kudrna et al. (2011); Shapoval and Lukhtanov (2015).) Considered here as ssp. of *Polyommatusdamone* (Eversmann, 1841).
Lukhtanov & Budashkin, 1993	
* Melitaea punica *	Distributed outside Europe in northern Africa (Toth et al. 2014).
Oberthür, 1876	
* Melitaea telona *	Distributed outside Europe in Levant (Toth et al. 2014).
Fruhstorfer, 1908	
* Pseudochazara mniszechii *	Distributed outside Europe in Asian part of Turkey (Hesselbarth et al. 1995). *P.tisiphone*, often considered as a subspecies of *P.mniszechii*, was shown not to be closely related to it (Verovnik and Wiemers 2016).
(Herrich-Schäffer, 1851)	
* Pseudochazara beroe *	Distributed outside Europe in Asian part of Turkey (Hesselbarth et al. 1995).
(Freyer, 1843)	

**Table 7.** List of the 14 European butterfly species that are affected by the gender agreement provision.

**Name**	**Original species epithet**
* Agriades pyrenaicus *	* pyrenaica *
* Carcharodus tripolinus *	* tripolina *
* Colias crocea *	* croceus *
* Cupido decoloratus *	* decolorata *
* Erebia aethiopella *	* aethiopellus *
* Erebia albergana *	* alberganus *
* Erebia montana *	* montanus *
* Erebia stiria *	* stirius *
* Hipparchia azorina *	* azorinus *
* Hyponephele lupina *	* lupinus *
* Kretania hesperica *	* hespericus *
* Lycaena dimorpha *	* dimorphus *
* Lycaena ottomana *	* ottomanus *
* Tarucus balkanicus *	* balkanica *

Compared to the last version 2.6.2 of Fauna Europaea, nine species have been excluded from the list (Table 6). On the other hand, 15 species were added to the list. Another recently discovered species, *Spialiarosae* Hernández-Roldán, Dapporto, Dincă, Vicente & Vila, 2016, has already been added to the Fauna Europaea database.

Apart from the changes due to the gender agreement provision (Table 7), only three species names had to be changed due to new nomenclatural evidence: *Pyrgusbellieri* (Oberthür, 1910) to *Pyrgusfoulquieri* (a name which had already been used in previous field guides), *Proterebiaafra* (Fabricius, 1787) to *Proterebiaphegea* (hopefully solving a longstanding controversy, see e.g., Jutzeler and Lafranchis 2011), and the mandatory change of *Pseudochazarahippolyte* (Esper, 1783) to *Pseudochazaramercurius* due to primary homonomy.

A larger number of changes concern the genus names. Most of them are in the family Lycaenidae, where 26 species changed their genus name, mainly based on the molecular study by Talavera et al. (2013), which substantially improved our knowledge of phylogenetic relationships of the subtribe Polyommatina. However, none of the genus names is new and many of them have already been used with the same species. In addition, four species formerly placed in the genus *Argynnis* were transferred into the genera *Fabriciana* and *Speyeria*, based on the study by De Moya et al. (2017). The former genus name had already been used previously for the same species, whereas the latter seems new to European lepidopterists, but is commonly used in North America. Although it could be argued that the change was avoidable by keeping a larger genus *Argynnis*, a solution originally also favoured by Simonsen et al. (2006), this would have meant to rename a large number of North American butterflies currently placed in the genus *Speyeria*, and was rejected by North American lepidopterists. Therefore, the recommended changes appear to cause the least changes on a global level and will hopefully contribute to a more consistent taxonomy of Holarctic Argynnini.

Finally, quite a number of minor changes have been implemented, which correct mistakes in names of authors, year of publication, or the incorrect use of parentheses for species that have changed generic combinations. An example is the change of year for 6 butterfly names due to a correction of the publication date of Linnaeus’ Fauna Svecica. Evenhuis (1997: 480) has shown convincingly that this edition was actually published on [14 November 1760], not “1761” as stated in the title page of the work and Bousquet (2016) also agrees with that year of publication.

## Conclusions

Taking into account the many recent research findings, especially those with molecular methods, we think that the new taxonomy represents a step forward in stabilizing European butterfly taxonomy and nomenclature. Nevertheless, we have to note that some groups, e.g., the genera *Euchloe*, *Callophrys*, *Pseudophilotes*, *Melitaea*, and *Hipparchia*, as well as the subgenus Agrodiaetus of the genus *Polyommatus* are still in need of revision, which will certainly lead to additional changes in the future. Furthermore, we still have large knowledge gaps for species in other regions of the Palearctic region (especially in Central Asia), which might require changes in order to achieve a consistent taxonomy of Palearctic and Holarctic butterflies.

## Appendix 1

Evidence that the junior synonym *Polyommatusottomanus* Lefèbvre, 1831 has been used to denote the taxon currently known as *Lycaenaottomana* (Lefèbvre, [1831]), in at least 25 works, published by at least 10 authors during the last 50 years and encompassing a span of not less than 10 years, and thus fulfilling the conditions of article 23.9.1.2 of the code in order to reverse the precedence of *Lycaenalegeri* Freyer, 1830.

Already during the decades immediately following the publication of *legeri* Freyer, this name does not seem to have been used but as a subjective junior synonym of *ottomanus* Lefèbvre. The latter name was thought to represent the valid name and was first used in its original combination (*Polyommatusottomanus*) and starting from the 20^th^ century mostly in the combination of *Chrysophanusottomanus*:

• Brullé (1832): *Polyommatusottomanus* Lef.

• Herrich-Schäffer (1843): *Polyomm. Ottomanus* Lef.; synonym: *Legeri*

• Mann (1862): *Polyommatusottomanus* Lef.

• Lang (1884): *PolyommatusOttomanus*, Lefebrve [sic]; synonym: *Legeri*, Frr.

• Rebel (1903): *ChrysophanusOttomanus* Lef.

• Spuler (1908): *Chrysóphanus ottománus* Lef.

• Courvoisier (1921): *Chrysophanusottomanus* Lefebvre 1830; synonym: *legeri* Freyer, 1832

• Galvagni (1924): *Chrysophanusottomanus* Lef.

• Rebel and Zerny (1934): *Chrysophanusottomanus* Lef.

• Kanus (1963): Heodes (Chrysophanus) ottomanus Lef.

During the last 50 years we are not aware of any use of *legeri* Freyer, except as a subjective junior synonym of *ottomanus* Lefèbvre. The latter name was mostly used in the combination of *Heodesottomanus* and later as *Lycaenaottomanus* or, due to the gender agreement rule of the code, as *Lycaenaottomana*:

1. Higgins and Riley (1970): *Heodesottomanus* Lefèbvre, 1830

2. Higgins (1975): *Heodesottomanus* Lefèbvre 1830

3. Higgins and Riley (1978): *Heodesottomanus* Lefèbvre 1830

4. Schmidt-Koehl (1980): *Heodesottomanus* Lefebvre, 1830

5. Krzywicki (1981): *Heodesottomanus* Lefevre [sic]

6. Wiemers (1983): *Heodesottomanusottomanus* Lef.

7. Higgins and Riley (1983): *Heodesottomanus* Lefèbvre, 1830

8. Kudrna (1986): *Lycaenaottomanus* Lefebvre, 1830

9. Jakšić (1988): *Lycaenaottomanus* Lefèbvre, 1830

10. Schaider and Jakšić (1989): *Lycaenaottomanus* Lef.

11. Hesselbarth et al. (1995): *Lycaenaottomana* (Lefebvre, [1830]); synonym: “Gen.IX. *Lycaena*. 182. Pap. *Legeri*” Freyer, C.F., [Dezember] 1830

12. Karsholt and Razowki (1996): *Lycaenaottomanus* (Lefèbvre, 1830)

13. Pamperis (1997): *Heodesottomanus*

14. Jakšić (1998): *Lycaenaottomanus* Lefèbvre, 1830

15. Tolman and Lewington (1998): *Lycaenaottomana* (Lefèbvre, 1830)

16. Abadjiev (2001): *Lycaenaottomana* (Lefebvre, [1830])

17. Bozano and Weidenhoffer (2001): *Lycaenaottomanus* (Lefebvre, 1830); synonym: *legeri* Freyer, 1839

18. Mihoci et al. (2005): *Lycaenaottomanus* (Lefèbvre, 1830)

19. Coutsis and Ghavalas (2006): *Lycaenaottomanus* (Lefebvre, 1830)

20. Wagener (2006): *Lycaenaottomanus* (Lefebvre, 1830)

21. Settele et al. (2008): *Lycaenaottomana* (Lefebvre, 1830)

22. Tolman and Lewington (2008): *Lycaenaottomana* Lefèbvre, 1830

23. Pamperis (2009): *Lycaenaottomanus*

24. Van Swaay et al. (2010): *Lycaenaottomana* (Lefèbvre, 1830)

25. Tshikolovets (2011): *Lycaenaottomana* (Lefebvre, [1830]); synonym: *legeri* Freyer, 1839

26. Kemal and Koçak (2011): Lycaena (Heodes) ottomanus (Lefèbvre, [1830]); synonym: *legeri* Freyer, 1830

27. Kudrna et al. (2011): *Lycaenaottomana* (Lefebvre, 1831)

28. Koren et al. (2012): *Lycaenaottomana* (Lefèbvre, 1830)

29. Verovnik & Popović (2012): *Lycaenaottomanus* (Lefèbvre, 1830)

30. Kudrna et al. (2015): *Lycaenaottomana* (Lefebvre, 1831)

31. Çalişkan (2016): *Lycaenaottomanus* (Lefèbvre, [1830])

**References**:

Abadjiev S (2001) An atlas of the Distribution of the Butterflies of Bulgaria (Lepidoptera: Hesperioidea & Papilionoidea). Zoocartographia Balcanica 1: 1–335.

Baytaş A (2007) A field guide to the butterflies of Turkey. NTV, Istanbul, 218 pp.

Bozano GC, Weidenhoffer Z (2001) Lycaenidae part I. Subfamily Lycaeninae. In: Bozano GC (Ed.) Guide to the butterflies of the Palearctic region. Omnes Artes, Milano, 62 pp.

Brullé M (1832) Expédition scientifique de Morée. Section des Sciences Physiques. Tome III. 1^re^ Partie – Zoologie. Deuxième Section – Des animaux articulé. FG Levrault, Strasbourg, 402 pp.

Çalişkan SS (2016) Contribution to the Butterfly Fauna of the Gevne Valley (South Turkey: West Toros) – Entomofauna 37: 281–296. https://www.zobodat.at/pdf/ENT_0037_0281-0296.pdf

Courvoisier LG (1921) Zur Synonymie des Genus *Lycaena*. Deutsche entomologische Zeitschrift Iris 35: 54–110. http://www.zobodat.at/pdf/Deutsche-ent-Z-Iris_35_0054-0110.pdf

Coutsis JG, Ghavalás N (2006) Butterflies and Skippers from the Greek island of Évvia (= Euboea) (Lepidoptera: Hesperioidea & Papilionoidea). Phegea 34(2): 49–55. http://www.phegea.org/Phegea/2006/Phegea34-2_49-55.pdf

Galvagni E (1935) Griechische Falter, insbesondere über die auf der Griechenlandreise der Universität Wien, Ostern 1933, 8.–23. April beobachteten Schmetterlinge. Schluß. Zeitschrift des Österreichischen Entomologischen Vereins 20: 5–7. https://www.zobodat.at/pdf/ZOEV_20_0005-0007.pdf

Herrich-Schäffer GAW (1843) Systematische Bearbeitung der Schmetterlinge von Europa, zugleich als Text, Revision und Supplement zu Jakob Hübner’s Sammlung europäischer Schmetterlinge. Bd. 1. Die Tagfalter. Manz, Regensburg, 164 pp.

Hesselbarth G, Van Oorschot H, Wagener S (1995) Die Tagfalter der Türkei unter Berücksichtigung der angrenzenden Länder. Author’s edition, Bocholt, 1354 + 847 pp.

Higgins LG, Riley ND (1970) A field guide to the butterflies of Britain and Europe. Collins, London & Glasgow, 381 pp.

Higgins LG (1975) The classification of European Butterflies. Collins, London & Glasgow, 320 pp.

Higgins LG, Riley ND (1978) Die Tagfalter Europas und Nordwestafrikas. Übersetzt und bearbeitet von Dr. Walter Forster. 2. Auflage. Paul Parey, Hamburg & Berlin, 377 pp.

Higgins LG, Riley ND (1983) A field guide to the butterflies of Britain and Europe (5^th^ edn). Collins, London, 384 pp.

Jakšić P (1988) Privremene Karte Rasprostranjenosti Dnevnih Leptira Jugoslavije / Provisional Distribution Maps of the Butterflies of Yugoslavia (Lepidoptera, Rhopalocera). Societas Entomologica Jugoslavica, Zagreb, 215 pp.

Jakšić P (1998) Male genitalia of butterflies on Balkan Peninsula with a checklist (Lepidoptera: Hesperioidea and Papilionoidea). František Slamka, Bratislava, 144 pp.

Kanus A (1963) Türkiye Lepidoptera Faunası İçin İlkel Liste. III. Bıtkı Koruma Bülteni 3: 83–85.

Karsholt O, Razowski J (Eds) (1996) The Lepidoptera of Europe. A Distributional Checklist. Apollo Books, Stenstrup, 380 pp.

Kemal M, Koçak AÖ (2011) A synonymical, and distributional checklist of the Papilionoidea and Hesperioidea of East Mediterranean countries, including Turkey (Lepidoptera). Priamus (Suppl.) 25: 1–162. [42 pls]

Koren T, Štih A, Trkov D, Črne M (2012) New records of Grecian Cooper, *Lycaenaottomana* (Lefèbvre, 1830) (Lep.: Lycaenidae) in Croatia. Entomologist’s Record and Journal of Variation 124: 215–223.

Krzywicki M (1981) Anmerkungen zur Tagfalterfauna Bulgariens. Nota lepidopterologica 4(1–2): 29–46. https://www.zobodat.at/pdf/Nota-lepidopterologica_4_0029-0046.pdf

Kudrna O (1986) Aspects of the Conservation of Butterflies in Europe. In: Kudrna O (Ed.) Butterflies of Europe, vol 8, Aula, Wiesbaden, 323 pp.

Kudrna O, Harpke A, Lux K, Pennerstorfer J, Schweiger O, Settele J, Wiemers M (2011) Distribution atlas of butterflies in Europe. Gesellschaft für Schmetterlingsschutz e.V., Halle, 576 pp.

Kudrna O, Pennerstorfer J, Lux K (2015) Distribution Atlas of European Butterflies and Skippers. Peks, Schwanfeld, 632 pp.

Lang HC (1884) Rhopalocera Europae Descripta et Delineata. The butterflies of Europe described and figured. L. Reeve, London, 396 pp. https://doi.org/10.5962/bhl.title.126361

Mann JJ (1862) Verzeichniss der im Jahre 1851 bei Brussa in Kleinasien gesammelten Schmetterlinge. Tafel 3. Wiener Entomologische Monatsschrift 6: 356–371. https://biodiversitylibrary.org/page/31455868

Mihoci I, Tvrtković N, Šašić M (2005) Grecian Copper *Lycaenaottomanus* (Lefèbvre, 1830) (Lepidoptera, Lycaenidae) – a new species in the Croatian butterfly fauna. Natura Croatica 14(4): 255–262. https://hrcak.srce.hr/file/2746

Pamperis LN (1997) The Butterflies of Greece. Bastas-Plessas, Athens, 559 pp.

Pamperis LN (2009) The Butterflies of Greece. 2^nd^ revised edition. Editions Pamperis, Lárisa, 766 pp.

Rebel H (1903) Studien über die Lepidopterenfauna der Balkanländer. 1. Teil: Bulgarien und Ostrumelien. Annalen des naturhistorischen Museums in Wien 18: 123–348.

Rebel H, Zerny H (1934) Wissenschaftliche Ergebnisse der im Auftrage und mit Kosten der Akademie der Wissenschaften in Wien im Jahre 1918 entsendeten Expedition nach Nordalbanien. Die Lepidopterenfauna Albaniens (mit Berücksichtigung der Nachbargebiete). Denkschriften der Akademie der Wissenschaften. Mathematisch Naturwissenschaftliche Klasse 103: 37–161.

Schaider P, Jakšić P (1989) Die Tagfalter von jugoslawisch Mazedonien. Paul Schaider, München, 82 pp. [46 pls, 199 maps]

Schmidt-Koehl W (1980) Geographisch-entomologische Studienreise nach Südgriechenland im Juli 1979. Atalanta 11: 212–233. https://www.zobodat.at/pdf/Atalanta_11_0212-0233.pdf

Settele J, Kudrna O, Harpke A, Kühn I, Van Swaay C, Verovnik R, Warren M, Wiemers M, Hanspach J, Hickler T, Kühn E, Van Halder I, Veling K, Vliegenthart A, Wynhoff I, Schweiger O (2008) Climatic Risk Atlas of European Butterflies. Pensoft, Sofia, Moscow. BioRisk 1 (Special Issue): 1–710. https://doi.org/10.3897/biorisk.1

Spuler A (1908) Die Schmetterlinge Europas. I. Band. Schweizerbart, Stuttgart, 385 pp.

Tolman T, Lewington R (1998) Die Tagfalter Europas und Nordwestafrikas. Übersetzt und bearbeitet von Matthias Nuss. Kosmos, Stuttgart, 319 pp.

Tolman T, Lewington R (2008) Collins Butterfly Guide. The most complete guide to the butterflies of Britain and Europe. HarperCollins, London, 384 pp.

Tshikolovets VV (2011) Butterflies of Europe and the Mediterranean Area. Tshikolovets Publications, Pardubice, 544 pp.

Van Swaay CAM, Cuttelod A, Collins S, Maes D, Munguira LM, Šašić M, Settele J, Verovnik R, Verstrael T, Warren M, Wiemers M, Wynhoff I (2010) European Red List of Butterflies. Publications Office of the European Union, Luxembourg, 47 pp. http://ec.europa.eu/environment/nature/conservation/species/redlist/downloads/European_butterflies.pdf

Verovnik R, Popović M (2012) Annotated checklist of Albanian butterflies (Lepidoptera, Papilionoidea and Hesperioidea). ZooKeys 323: 75–89. https://doi.org/10.3897/zookeys.323.5684

Wagener PS (2006) Butterfly Diversity and Protection in Turkey. Bonner Zoologische Beiträge 54(1): 3–23. https://www.zobodat.at/pdf/Bonner-Zoologische-Beitraege_54_0003-0023.pdf

Wiemers M (1983) Tagfalterbeobachtungen in Mittelgriechenland im August 1982. Nachrichten des entomologischen Vereins Apollo, Neue Folge 4(2): 25–58. https://www.zobodat.at/pdf/NEVA_4_0025-0058.pdf

## Supplementary materials

Supplementary material 1 Distributional checklist of European butterflies (country checklist) Data type: occurrence This is an open access article distributed under the terms of the Creative Commons Attribution License (CC BY 4.0), which permits unrestricted use, distribution, and reproduction in any medium, provided the original author and source are credited.Martin Wiemers, Emilio Balletto, Vlad Dincă, Zdenek Faltynek Fric, Gerardo Lamas, Vladimir Lukhtanov, Miguel L. Munguira, Chris A. M. van Swaay, Roger Vila, Albert Vliegenthart, Niklas Wahlberg, Rudi Verovnik

Supplementary material 2 Distributional checklist of European butterflies (CoL) Data type: occurrence This is an open access article distributed under the terms of the Creative Commons Attribution License (CC BY 4.0), which permits unrestricted use, distribution, and reproduction in any medium, provided the original author and source are credited.Martin Wiemers, Emilio Balletto, Vlad Dincă, Zdenek Faltynek Fric, Gerardo Lamas, Vladimir Lukhtanov, Miguel L. Munguira, Chris A. M. van Swaay, Roger Vila, Albert Vliegenthart, Niklas Wahlberg, Rudi Verovnik

## References

[B1] Achtelik G (2006) Molekularbiologische Analyse der genetischen Diversität des *Melitaeaathalia/celadussa*-Komplexes (Lepidoptera: Nymphalidae) unter Anwendung der ISSR-PCR auf Art-, Unterart- und Populationsebene. Dissertation, University of Bochum, 165 pp https://hss-opus.ub.ruhr-uni-bochum.de/opus4/frontdoor/index/index/docId/3119

[B2] Albre J Gers C Legal L (2008) Molecular phylogeny of the *Erebiatyndarus* (Lepidoptera, Rhopalocera, Nymphalidae, Satyrinae) species group combining CoxII and ND5 mitochondrial genes: A case study of a recent radiation. Molecular Phylogenetics and Evolution 47: 196–210. 10.1016/j.ympev.2008.01.009 18295512

[B3] Balletto E Bonelli S (2014) Case 3637 *Papiliophoebus* De Prunner, 1798: proposed conservation in its accustomed usage by suppression of *Papiliophoebus* Fabricius, 1793 (Insecta, Lepidoptera, Papilionidae). Bulletin of Zoological Nomenclature 71(2): 75–80. 10.21805/bzn.v71i2.a6

[B4] Balletto E Cassulo LA Bonelli S (2014) An annotated Checklist of the Italian Butterflies and Skippers (Papilionoidea, Hesperiioidea). Zootaxa 3853(1): 1–114. 10.11646/zootaxa.3853.1.1 25284503

[B5] Beccaloni G Scoble M Kitching I Simonsen T Robinson G Pitkin B Hine A Lyal C [Eds] (2003) The Global Lepidoptera Names Index (LepIndex). http://www.nhm.ac.uk/our-science/data/lepindex/lepindex/ [last accessed 25 April 2018]

[B6] Bousquet Y (2016) Litteratura Coleopterologica (1758–1900): a guide to selected books related to the taxonomy of Coleoptera with publication dates and notes. ZooKeys 583: 1–776. 10.3897/zookeys.583.7084

[B7] Coutsis JG (2005) Revision of the *Turananaendymion* species-group (Lycaenidae). Nota lepidopterologica 27(4): 251–272. https://biodiversitylibrary.org/page/46956733

[B8] Coutsis JG (2017) A re-evaluation of certain generic transfers of species-group taxa belonging to the subtribes Polyommatiti and Leptotiti (Lepidoptera: Lycaenidae, Polyommatini). Phegea 45(2): 26–34. http://www.phegea.org/Phegea/Phegea45_2017.htm

[B9] Coutsis JG van Oorschot H (2011) Differences in the male and female genitalia between *Iphiclidespodalirius* and *Iphiclidesfeisthamelii*, further supporting species status for the latter (Lepidoptera: Papilionidae). Phegea 39(1): 12–22. https://biodiversitylibrary.org/page/49125235

[B10] Cowan CF (1970) Boisduval’s Icones des Lèpidoptéres d’Europe “1832” [-1841]. Journal of the Society for the Bibliography of Natural History 5(4): 291–302. 10.3366/jsbnh.1970.5.PART_4.291

[B11] D’Aguilar J Raimbault F (1990) Notes de bibliographie entomologique. 3. Geoffroy, Fourcroy et l’article 51 du Code de Nomenclature. L’Entomologiste 46(1): 37–40. https://lentomologiste.fr/wp-content/uploads/1990-46/lentomologiste_1990_46_1.pdf

[B12] Dapporto L (2009) Speciation in Mediterranean refugia and post-glacial expansion of *Zerynthiapolyxena* (Lepidoptera, Papilionidae). Journal of zoological systematics and evolutionary research 48(3): 229–237. 10.1111/j.1439-0469.2009.00550.x

[B13] de Jong Y Verbeek M Michelsen V Bjørn P Los W Steeman F Bailly N Basire C Chylarecki P Stloukal E Hagedorn G Wetzel F Glöckler F Kroupa A Korb G Hoffmann A Häuser C Kohlbecker A Müller A Güntsch A Stoev P Penev L (2014) Fauna Europaea – all European animal species on the web. Biodiversity Data Journal 2: e4034. 10.3897/BDJ.2.e4034 PMC4206781 25349527

[B14] De Moya RS Savage WK Tenney C Bao X Walberg N Hill RI (2017) Interrelationships and diversification of *Argynnis* Fabricius and *Speyeria* Scudder butterflies. Systematic Entomology 42: 635–649. 10.1111/syen.12236

[B15] Dincă V Montagud S Talavera G Hernández-Roldán JL Munguira ML García-Barros E Hebert PDN Vila R (2015) DNA barcode reference library for Iberian butterflies enables a continental-scale preview of potential cryptic diversity. Scientific Reports 5: 12395. 10.1038/srep12395 PMC4513295 26205828

[B16] Dincă V Wiklund C Lukhtanov VA Kodandaramaiah U Norén K Dapporto L Wahlberg N Vila R Friberg M (2013) Reproductive isolation and patterns of genetic differentiation in a cryptic butterfly species complex. Journal of Evolutionary Biology 26(10): 2095–2106. 10.1111/jeb.12211 23909947 PMC4413813

[B17] Dincă V Dapporto L Vila R (2011a) A combined genetic-morphometric analysis unravels the complex biogeographical history of *Polyommatusicarus* and *Polyommatuscelina* Common Blue butterflies. Molecular Ecology 20(18): 3921–3935. 10.1111/j.1365-294X.2011.05223.x 21883579

[B18] Dincă V Lukhtanov VA Talavera G Vila R (2011b) Unexpected layers of cryptic diversity in wood white *Leptidea* butterflies. Nature Communications 2: 324. 10.1038/ncomms1329 21610727

[B19] Dumont D (2004) Révision du genre *Iolana* Bethune-Baker 1914 (Lepidoptera: Lycaenidae). Linneana belgica 19(8): 332–358.

[B20] Espeland M Breinholt J Willmott KR Warren AD Vila R Toussaint EFA Maunsell SC Aduse-Poku K Talavera G Eastwood R Jarzyna MA Guralnick R Lohman DJ Pierce NE Kawahara AY (2018) A comprehensive and dated phylogenomic analysis of butterflies. Current Biology 28(5): 770–778. 10.1016/j.cub.2018.01.061 29456146

[B21] Evenhuis NL (1997) Litteratura Taxonomica Dipterorum (1758–1930). Backhuys Publishers, Kerkwerve, NL, 871 pp.

[B22] Ganglbauer L Heyden L (1906) Über die Entomologia parisiensis von Geoffroy und Fourcroy. Wiener Entomologische Zeitung 25(7/8): 301–392. 10.5962/bhl.part.5406

[B23] Grieshuber J Worthy B Lamas G (2012) The genus *Colias* Fabricius, 1807. Jan Haugum’s annotated catalogue of the Old World *Colias* (Lepidoptera, Pieridae). Tshikolovets Publications, Pardubice-Bad Griesbach-Caretham-Lima, 438 pp.

[B24] Griffin FJ (1931) On the Dates of the Parts of “Meigen (J. W.) Syst. Besch. Eur. Schmett.”. Annals and Magazine of Natural History, London 8: 421.

[B25] Gross FJ (1973) *Satyrussintenisi* auch in Europa, nebst Beschreibung einer neuen Unterart (Lep., Satyridae). Entomologische Zeitschrift 83: 211–214.

[B26] Hanus J Thèye M-L (2010) *Parnassiusphoebus* (Fabricius, 1793), a misidentified species (Lepidoptera: Papilionidae). Nachrichten des entomologischen Vereins Apollo, Neue Folge 31(1/2): 71–84. https://www.zobodat.at/pdf/NEVA_31_0071-0084.pdf

[B27] Heikkilä M Kaila L Mutanen M Peña C Wahlberg N (2012) Cretaceous origin and repeated tertiary diversification of the redefined butterflies. Proceedings of the Royal Society Biological Sciences 279(1731): 1093–1099. 10.1098/rspb.2011.1430 21920981 PMC3267136

[B28] Hemming AF (1937) Hübner. A bibliographical and systematic account of the entomological works of Jacob Hübner. Royal Entomological Society, London, 605 pp [Vol. 1], 274 pp [Vol. 2].

[B29] Hemming AF (1967) The generic names of the butterflies and their type-species (Lepidoptera: Rhopalocera). Bulletin of the British Museum (Natural History). Entomology Suppl. 9: 1–509.

[B30] Heppner JB (1981) The dates of E. J. C. Esper’s “Die Schmetterlinge in Abbildungen.... “ 1776–[1830]. Archives of Natural History 10(2): 251–254. 10.3366/anh.1981.10.2.251

[B31] Heppner JB (1982) Dates of selected Lepidoptera literature for the western hemisphere fauna. Journal of the Lepidopterists’ Society 36(2): 87–111. http://images.peabody.yale.edu/lepsoc/jls/1980s/1982/1982-36(2)87-Heppner.pdf

[B32] Hernández-Roldán JL Vicente JC Vila R Munguira ML (2018) Natural history and immature stage morphology of *Spialia* Swinhoe, 1912 in the Iberian Peninsula (Lepidoptera, Hesperiidae). Nota Lepidopterologica 41(1): 1–22. 10.3897/nl.41.13539

[B33] Hernández-Roldán JL Dapporto L Dincă V Vicente JC Hornett EA Šíchová J Lukhtanov V Talavera G Vila R (2016) Integrative analyses unveil speciation linked to host plant shift in *Spialia* butterflies. Molecular Ecology 25(17): 4267–84. 10.1111/mec.13756 27393640

[B34] Hesselbarth G Van Oorschot H Wagener S (1995) Die Tagfalter der Türkei unter Berücksichtigung der angrenzenden Länder. Author’s edition, Bocholt, 1354 + 847 pp.

[B35] Higgins LG Riley ND (1970) A field guide to the butterflies of Britain and Europe. Collins, London & Glasgow, 381 pp.

[B36] ICZN (1999) International Code of Zoological Nomenclature. Fourth Edition. International Trust for Zoological Nomenclature, London, 306 pp.

[B37] ICZN (2017) Opinion 2382 (Case 3637) – Conservation of the accustomed usage of *Papiliophoebus* De Prunner, 1798 by suppression of *Papiliophoebus* Fabricius, 1793 not approved (Insecta, Lepidoptera, Papilionidae). Bulletin of Zoological Nomenclature 73(2–4): 148–149. http://www.bioone.org/doi/abs/10.21805/bzn.v73i2.a21

[B38] Jutzeler D Lafranchis T (2011) Die Larvalstadien von *Proterebiaafrapyramus* (De Louker & Dils, 1987) aus dem Norden Griechenlands. Larvalentwicklung der dalmatinischen *P.afradalmata* (Godart, 1824) im Vergleich und zur Geschichte des Namens unserer Art (Lepidoptera: Nymphalidae, Satyrinae). Entomologica romanica 16: 5–18. http://er.lepidoptera.ro/16_2011/ER16201101_Jutzeler_Lafranchis.pdf

[B39] Karsholt O Nielsen ES (1986) The Lepidoptera described by C. P. Thunberg. Entomologica scandinavica 16(4): 433–463. 10.1163/187631285X00388

[B40] Karsholt O Nieukerken EJ van (Eds) (2011) Lepidoptera Fauna Europaea version 2.4. http://www.faunaeur.org [online 28 January 2011].

[B41] Karsholt O Razowski J (Eds) (1996) The Lepidoptera of Europe. A Distributional Checklist. Apollo Books, Stenstrup, 380 pp.

[B42] Koçak AÖ (1981) On the type-species of the genus *Proterebia* Roos & Arnscheid, 1980 (Satyridae, Lepidoptera). Priamus 1(1): 6–7. https://archive.org/details/CentreForEntomologicalStudiesAnkaraPriamus11/page/n1

[B43] Kudrna O (2015) The never ending story of Schiffermüller’s names – a long evaded nomenclatural issue of pressing urgency and a special case for the ICZN (Insecta: Lepidoptera). Quadrifina 12: 17–26.

[B44] Kudrna O Belicek J (2005) On the ‘Wiener Verzeichnis’, its authorship and the butterflies named therein. Oedippus 23: 1–32. http://www.ufz.de/export/data/10/129759_Oedippus_23.pdf

[B45] Kudrna O Harpke A Lux K Pennerstorfer J Schweiger O Settele J Wiemers M (2011) Distribution atlas of butterflies in Europe. Gesellschaft für Schmetterlingsschutz e. V., Halle, 576 pp.

[B46] Lafranchis T Delmas S Mazel R (2015) Le contact *Iphiclidesfeisthamelii - I.podalirius*. Statut de ces deux taxons (Lepidoptera, Papilionidae). Revue de l’Association Roussillonnaise d’Entomologie 24(3): 111–132. http://diatheo.weebly.com/uploads/2/8/2/3/28235851/feisthamelii_podalirius_lafranchis_2015__2_.pdf

[B47] Lamas G (2013) *Papiliolachesis* Hübner, 1790 has priority over *Papilionemausiaca* Esper, [1793] (Lepidoptera: Nymphalidae, Satyrinae). SHILAP Revista de lepidopterologia 41(162): 207–211. http://citeseerx.ist.psu.edu/viewdoc/download?doi=10.1.1.910.9054&rep=rep1&type=pdf

[B48] Lefèbvre A (1831) *Polyommatusottomanus*. Latreille. Magasin de zoologie, d’Anatomie comparée et de la Paléontologie 1(2): 19–20. [Paris]

[B49] Lempke BJ (1949) Rebel’s edition of Berge’s “Schmetterlingsbuch”. Journal of the Society for the Bibliography of Natural History 2(5): 171–172. 10.3366/jsbnh.1949.2.5.171

[B50] Leneveu J Chichvarkhin A Wahlberg N (2009) Varying rates of diversification in the genus *Melitaea* (Lepidoptera: Nymphalidae) during the past 20 million years. Biological Journal of the Linnean Society 97(2): 346–361. 10.1111/j.1095-8312.2009.01208.x

[B51] Lorković Z (1969) Karyologischer Beitrag zur Frage der Fortpflanzungsverhältnisse südeuropäischer Taxone von *Pierisnapi* (L.) (Lep., Pieridae). Biolośki Glasnik 21(1968): 95–136.

[B52] Lukhtanov VA (2000) Zur Systematik und Verbreitung der Taxa der *Athamanthiadimorpha* – Gruppe. Atalanta 31(1/2): 179–192. http://www.zobodat.at/pdf/Atalanta_31_0179-0192.pdf

[B53] Lukhtanov VA Pelham JP Cotton AM Calhoun JV (in press) Case 3767: *Papiliophoebus* Fabricius, 1793 (currently *Parnassiusphoebus*) (Insecta, Lepidoptera): proposed conservation of prevailing usage of the specific name and that of *Doritisariadne* Lederer, 1853 (currently *Parnassiusariadne*) by the designation of a neotype. Bulletin of Zoological Nomenclature. [accepted 2 October, 2018]

[B54] Lvovsky AL Morgun DV (2007) Butterflies of the Eastern Europe. KMK Scientific Press, Ltd., Moscow, 443 pp. [In Russian]

[B55] Maes D, Verovnik R, Wiemers M, Brosens D, Beshkov S, Bonelli S, Buszko J, Cantú Salazar L, Cassar LF, Collins S, Dincă V, Djuric M, Dusej G, Elven H, Franeta F, Garcia Pereira P, Geryak Y, Goffart P, Gór A, Hiermann U, Höttinger H, Huemer P, Jakšić P, John E, Kalivoda H, Kati V, Komac B, Kőrösi A, Kulak AV, Kuussaari M, L’Hoste L, Lelo S, Mestdagh X, Micevski N, Mihut S, Monasterio León Y, Munguira ML, Murray T, Nielsen PS, Ólafsson E, Õunap E, Pamperis L, Pavlíčko A, Pettersson LB, Popov S, Popović M, Ryrholm N, Šašić M, Pöyry J, Savenkov N, Settele J, Sielezniew M, Sinev S, Stefanescu C, Švitra G, Tammaru T, Tiitsaar A, Tzirkalli E, Tzortzakaki O, van Swaay CAM, Viborg AL, Wynhoff I, Zografou K, Warren MS (submitted) Integrating national Red Lists for prioritising conservation actions for European butterflies. Journal of Insect Conservation. [in review]

[B56] Muñoz Sariot MG (2011) Biología y ecología de los licénidos españoles (Lepidoptera, Lycaenidae). M.G. Muñoz Sariot, 384 pp.

[B57] Mutanen M Wahlberg N Kaila L (2010) Comprehensive gene and taxon coverage elucidates radiation patterns in moths and butterflies. Proceedings of the Royal Society Biological Sciences 277(1695): 2839–2848. 10.1098/rspb.2010.0392 20444718 PMC2981981

[B58] Nekrutenko YP (1985) New blue butterfly taxa (Lepidoptera, Lycaenidae) from Transcaucasia and Middle Asia. Vestnik Zoologii 4: 29–35. [In Russian]

[B59] Olivier A (2000) Christian Friedrich Freyer’s “Neuere Beiträge zur Schmetterlingskunde mit Abbildungen nach der Natur”: an analysis, with new data on its publication dates (Insecta, Lepidoptera). Beiträge zur Entomologie 50(2): 407–486. https://www.zobodat.at/pdf/Beitraege-zur-Entomologie_50_0407-0486.pdf

[B60] Oorschot H Van Coutsis JG (2014) The genus *Melitaea* Fabricius, 1807 – Taxonomy and systematics with special reference to the male genitalia (Lepidoptera: Nymphalidae, Nymphalinae). Tshikolovets Publications, Pardubice, 360 pp.

[B61] PESI (2018) Pan-European Species directories Infrastructure. http://www.eu-nomen.eu/portal [2018-04-25]

[B62] Poche F (1938) Über die Erscheinungszeit und den Inhalt mehrerer Hefte, die bibliographische Anordnung und die verschiedenen Ausgaben von E. J. C. Esper, Die Schmetterlinge in Abbildungen nach der Natur mit Beschreibungen. Festschrift Embrik Strand (Riga) 4: 453–463.

[B63] Rebel H (1914) Vorweisung von Belegematerial über die Alveus- und Malvae- Gruppe der Gattung *Hesperia*. (Versammlung der Sektion für Lepidopterologie). Verhandlungen der kaiserlich-königlichen Zoologisch-Botanischen Gesellschaft Wien 64: 189–201.

[B64] Reiner J Hohenwarth S von (1792) Botanische Reisen nach einigen Oberkärntnerischen und benachbarten Alpen unternommen, und nebst einer ausführlichen Alpenflora und entomologischen Beiträgen als ein Handbuch für reisende Liebhaber. Erste Reise im Jahr 1791, C. F. Walliser, Klagenfurt, 270 pp [6 pls] https://www.biodiversitylibrary.org/item/84327

[B65] Sattler K Tremewan WG (1984) The Lepidoptera names of Denis & Schiffermüller – a case for stability. Nota lepidopterologica 7(3): 282–285. http://biostor.org/reference/117914

[B66] Sattler K Tremewan WG (2009) The authorship of the so-called ‘Wiener Verzeichnis’. Nota lepidopterologica 32(1): 3–10. http://biostor.org/reference/234732

[B67] Savela M (2018) Lepidoptera and some other life forms. Online Database. http://www.nic.funet.fi/pub/sci/bio/life/insecta/lepidoptera/ [last accessed on 25 April 2018]

[B68] Schmitt T Louy D Zimmermann E Habel JC (2016) Species radiation in the Alps: multiple range shifts caused diversification in Ringlet butterflies in the European high mountains. Organisms Diversity & Evolution 16(4): 791–808. 10.1007/s13127-016-0282-6

[B69] Seitz A (1907–1909) : Die Groß-Schmetterlinge der Erde. 1, 1 Die Palaearktischen Tagfalter.Fritz Lehmann’s Verlag, Stuttgart.

[B70] Shapoval N Lukhtanov V (2015) Taxonomic position and status of Polyommatus (Agrodiaetus) iphigenia (Lepidoptera, Lycaenidae) from the Peloponnese, southern Greece. Folia Biologica (Kraków) 63: 295–300. 10.3409/fb63_4.295 26975145

[B71] Simonsen TJ (2006a) Fritillary phylogeny, classification, and larval host plants: reconstructed mainly on the basis of male and female genitalic morphology (Lepidoptera: Nymphalidae: Argynnini). Biological Journal of the Linnean Society 89: 627–673. 10.1111/j.1095-8312.2006.00697.x

[B72] Simonsen TJ (2006b) Glands, muscles and genitalia. Morphological and phylogenetic implications of histological characters in the male genitalia of Fritillary butterflies (Lepidoptera: Nymphalidae: Argynnini). Zoologica Scripta 35: 231–241. 10.1111/j.1463-6409.2006.00225.x

[B73] Simonsen TJ Wahlberg N Brower AVZ de Jong R (2006) Morphology, molecules and fritillaries: approaching a stable phylogeny for Argynnini (Lepidoptera: Nymphalidae). Insect Systematics & Evolution 37: 405–418. 10.1163/187631206788831407

[B74] Talavera G Lukhtanov VA Pierce NE Vila R (2013) Establishing criteria for higher-level classification using molecular data: the systematics of *Polyommatus* blue butterflies (Lepidoptera, Lycaenidae). Cladistics 29(2): 166–192. 10.1111/j.1096-0031.2012.00421.x 34818826

[B75] Tatarinov AG Gorbunov PYu (2015) Spatial organisation of the Ural butterfly fauna (Lepidoptera: Papilionoidea & Hesperioidea). Fauna of the Urals and Siberia 1: 48–76. https://cyberleninka.ru/article/v/prostranstvennaya-organizatsiya-fauny-bulavousyh-cheshuekrylyh-lepidoptera-papilionoidea-hesperioidea-urala

[B76] Tolman T Lewington R (2008) Collins Butterfly Guide. The most complete guide to the butterflies of Britain and Europe. HarperCollins, London, 384 pp.

[B77] Toth JP Bereczki J Varga Z Rota J Sramko G Wahlberg N (2014) Relationships within the *Melitaeaphoebe* species group (Lepidoptera: Nymphalidae): new insights from molecular and morphometric information. Systematic Entomology 39: 749–757. 10.1111/syen.12083

[B78] Tremewan WG (1988) C. F. Freyer’s Neuere Beiträge zur Schmetterlingskunde mit Abbildungen nach der Natur. Bulletin of the British Museum (Natural History), Historical series 16(1): 1–16.

[B79] Tshikolovets VV (2011) Butterflies of Europe and the Mediterranean Area. Tshikolovets Publications, Pardubice, 544 pp.

[B80] Tsvetkov EV (2006) On two species of genus *Oeneis* Hübner, 1819 (Lepidoptera: Satyridae) from the Polar Urals. Eversmannia 5: 11–14. http://eversmannia.entomology.ru/eversmannia_05_11.pdf

[B81] van Nieukerken E Kaila L Kitching I Kristensen NP Lees D Minet J Mitter J Mutanen M Regier J Simonsen T et al. (2011) Order Lepidoptera Linnaeus, 1758. Zootaxa 3148: 212–221. http://www.mapress.com/zootaxa/2011/f/zt03148p221.pdf

[B82] Van Swaay CAM Cuttelod A Collins S Maes D Munguira LM Šašić M Settele J Verovnik R Verstrael T Warren M Wiemers M Wynhoff I (2010) European Red List of Butterflies. Publications Office of the European Union, Luxembourg, 47 pp http://ec.europa.eu/environment/nature/conservation/species/redlist/downloads/European_butterflies.pdf

[B83] Verovnik R Wiemers M (2016) Species delimitation in the Grayling genus *Pseudochazara* (Lepidoptera, Nymphalidae, Satyrinae) supported by DNA barcodes. ZooKeys 600: 131–154. 10.3897/zookeys.600.7798 PMC4926685 27408604

[B84] Vila R Lukhtanov VA Talavera G Gil-T F Pierce NE (2010) How common are dot-like distribution ranges? Taxonomical oversplitting in Western European *Agrodiaetus* (Lepidoptera, Lycaenidae) revealed by chromosomal and molecular markers. Biological Journal of the Linnean Society 101: 130–154. 10.1111/j.1095-8312.2010.01481.x

[B85] Vishnevskaya MS Saifitdinova AF Lukhtanov VA (2016) Karyosystematics and molecular taxonomy of the anomalous blue butterflies (Lepidoptera, Lycaenidae) from the Balkan Peninsula. Comparative Cytogenetics 10(5): 1–85. 10.3897/CompCytogen.v10i5.10944 PMC5220643 28105291

[B86] Warren BCS (1926) Monograph of the tribe Hesperiidi (European species) with revised classification of the subfamily Hesperiinae (Palaearctic species) based on the genital armature of the males. Transactions of the entomological Society of London 74: 1–170. [plts 1–60, 2 figs]

[B87] Welter-Schultes FW (2013) Guidelines for the capture and management of digital zoological names information, version 1.1, released on March 2013. Global Biodiversity Information Facility, Copenhagen, 126 pp http://www.gbif.org/orc/?doc_id=2784

[B88] Wiemers M Gottsberger B (2010) Discordant patterns of mitochondrial and nuclear differentiation in the Scarce Swallowtail *Iphiclidespodaliriusfeisthamelii* (Duponchel, 1832) (Lepidoptera: Papilionidae). Entomologische Zeitschrift 120(3): 111–115.

[B89] Wiemers M Stradomsky BV Vodolazhsky DI (2010) A molecular phylogeny of *Polyommatus* s. str. and *Plebicula* based on mitochondrial COI and nuclear ITS2 sequences. European Journal of Entomology 107: 325–336. 10.14411/eje.2010.041

[B90] Zinetti F Dapporto L Vovlas A Chelazzi G Bonelli S Balletto E Ciofi C (2013) When the rule becomes the exception. No evidence of gene flow between two *Zerynthia* cryptic butterflies suggests the emergence of a new model group. PLoS ONE 8(6): e65746. 10.1371/journal.pone.0065746 PMC3675026 23755277

